# Microbial succession in the gastrointestinal tract of dairy cows from 2 weeks to first lactation

**DOI:** 10.1038/srep40864

**Published:** 2017-01-18

**Authors:** Kimberly A. Dill-McFarland, Jacob D. Breaker, Garret Suen

**Affiliations:** 1 Department of Bacteriology, University of Wisconsin-Madison, Madison, Wisconsin, USA

## Abstract

Development of the dairy calf gastrointestinal tract (GIT) and its associated microbiota are essential for survival and milk production, as this community is responsible for converting plant-based feeds into accessible nutrients. However, little is known regarding the establishment of microbes in the calf GIT. Here, we measured fecal-associated bacterial, archaeal, and fungal communities of dairy cows from 2 weeks to the middle of first lactation (>2 years) as well as rumen-associated communities from weaning (8 weeks) to first lactation. These communities were then correlated to animal growth and health. Although succession of specific operational taxonomic units (OTUs) was unique to each animal, beta-diversity decreased while alpha-diversity increased as animals aged. Calves exhibited similar microbial families and genera but different OTUs than adults, with a transition to an adult-like microbiota between weaning and 1 year of age. This suggests that alterations of the microbiota for improving downstream milk production may be most effective during, or immediately following, the weaning transition.

Dairy products represent a significant industry worldwide^1^. Over 99% of this milk is produced by ruminants^1^, and like most herbivores, these animals rely solely on their gastrointestinal tract (GIT) microbial communities for the conversion of dietary plant substrates into accessible nutrients^2^. In ruminants, the majority of dietary breakdown occurs in the rumen, an enlarged foregut compartment where a diverse community of mainly bacteria and fungi ferment plant-derived lignocellulosic material and other substrates into short chain organic acids (SCOAs)^2^. These SCOAs are then absorbed by the host and contribute to animal growth and maintenance^3^ as well as milk production^4^. In addition to nutrition, the GIT microbiota also plays important roles in host development. For example, the native microbiota impacts the immune system by acting as a barrier against invading pathogens^5^ as well as promoting the proliferation of immune cells^6^,^7^. Moreover, SCOAs are required for rumen papillae formation^8^, and these compounds are predominately derived by microbial fermentation in the GIT^2^. Such development is critical to the host’s success after weaning^9^,^10^; however, little is known regarding the acquisition of GIT microorganisms or how differences in these communities may affect development.

While the GIT is thought to be sterile *in utero*, bacteria can be detected in the feces of calves as early as 12 hours after birth^11^, and are consistently found in the GIT pre-weaning^12^,^13^,^14^,^15^,^16^,^17^. Facultative anaerobes such as *Streptococcus* and *Enterococcus* are known early colonizers^15^ that serve to convert the GIT to a fully anaerobic environment^18^. Strictly anaerobic bacteria then establish and dominate the community within the first few days after birth^15^,^19^. In contrast, anaerobic fungi and methanogenic archaea do not appear in the rumen until approximately a week after birth^19^. Thus, colonization of the calf GIT begins very early in life, perhaps even during the birthing process as in other mammals^20^, but is a dynamic process with considerable fluctuation during the early life history of the animal.

After GIT colonization, the calf microbiota must still undergo considerable transformation as animals are weaned (6 to 12 weeks) and develop to reproductive maturity (1 year). The majority of studies have investigated GIT communities in calves up to or slightly beyond weaning^11^,^12^,^14^,^16^,^17^. While the gut microbiota is more adult-like at weaning than at birth, considerable differences exist between post-weaning calves and lactating cows^15^, indicating a need for assessment beyond the weaning transition. Two studies have compared the GIT microbiota of calves from birth to 6 months^13^ or 2 years of age^15^, but both reports compared different individuals at different ages. With the known high levels of inter-animal variation in calves^15^ and cows^21^, it is difficult to conclude when a fully adult microbiota became established in these animals. Given that the dairy cow microbiota is correlated to milk production efficiency^22^,^23^, and the difficulty in altering the established microbiota in adult cows^24^, assessment of the GIT microbiota in the same individual from calf to cow is needed to determine when alterations to the microbiota would be most effective to potentially improve downstream milk production.

Previous work has also primarily focused on the bacterial microbiota in calves. In the GIT, fungi are thought to be the first colonizers to physically attach to fibrous substrates and release substrates from the plant cell wall’s lignin matrix^25^. Methanogenic archaea can also alter the overall metabolic flux by siphoning off bacterial by-products, like hydrogen, and releasing carbon from the system in the form of methane^26^. Both outcomes can have considerable effects on the host by altering the concentrations and molar proportions of available SCOAs, and these communities need to be measured in order to fully understand the microbiota’s influences on the host.

To address these gaps in knowledge, we utilized next-generation sequencing to periodically track fecal microbiotas of developing dairy calves from two weeks of age until full maturity as lactating cows (>2 years) as well as rumen microbiotas from weaning (8 weeks) to maturity. These communities were then correlated to both growth and health metrics of calves and cows. To gain a more complete picture of the GIT microbiota, we specifically targeted bacterial, archaeal, and fungal communities, which are known to interact syntrophically to degrade and ferment plant-derived lignocellulosic material into available SCOAs (25, 26).

## Results

### Sequencing coverage

In this study, 3 bulls and 12 cows were retained through weaning (8 weeks) or first lactation cycle (>2 years), respectively. Feces were sampled from animals from 2 weeks to their first lactation (>2 years), while ruminal contents were sampled from bulls sacrificed at weaning and a subset of cannulated cows at 1 and 2 years. A fecal sample was not obtained at 2 weeks from 1 calf due to severe scours and a replacement sample was taken at 3 weeks. Feces were not obtained at 8 weeks from 4 calves due to on-farm scheduling conflicts, and replacements were not taken as animals were on a different diet by 9 weeks (Supplementary Table 1). All other fecal (N = 64) and all rumen (N = 54) samples were obtained at their target age (Supplementary Table 2).

Archaeal and bacterial amplicons were sequenced for all rumen and fecal samples. Fungal amplicons were sequenced for all rumen samples as well as 1- and 2-year feces. Calf samples failed to yield measurable fungal PCR products after extensive PCR optimization, and therefore, were not sequenced. After filtering in mothur, 200,000 (mean 1,700 ± 166 SE per sample) high-quality archaeal, 4.8 million (40,000 ± 1,900) high-quality bacterial, and 1.8 million (23,000 ± 2,700) high-quality fungal sequences were obtained. Sequence coverage was deemed sufficient, as determined by a Good’s coverage greater than 97% for all bacterial and fungal communities and most archaeal communities. A total of 10 fecal archaeal communities (2-week: 8, 4-week: 2) had low Good’s coverage (0–93%) after repeated sequencing yielded a minimum of 36,000 raw contigs per sample (Supplementary Table 3).

### Rumen and fecal communities are consistent with known cow microbiota

A total of 315 archaeal, 18,244 bacterial, and 1,485 fungal OTUs binned at 97% similarity were identified in the dataset. Archaeal communities ranged from 0 to 35, bacterial from 50 to 1,727, and fungal from 10 to 75 OTUs per sample after normalization (Supplementary Table 3). Archaeal communities contained >99% Euryarchaeota mostly within the genus *Methanobrevibacter*. Bacterial communities were dominated by the phyla Bacteroidetes and Firmicutes with smaller contributions from the Actinobacteria, Proteobacteria, Spirochaetes, Tenericutes, and Fibrobacteres. Within these phyla, the most abundant bacterial families were Bacteroidaceae, Lachnospiraceae, Prevotellaceae, Ruminococcaceae, Succinivibrionaceae, and Veillonellaceae. All fungal communities were dominated by the family Neocallimastigaceae or OTUs unclassified at the phylum level (Supplementary Table 4).

### Gut communities show higher alpha-diversity but lower beta-diversity with age

**Figure 1:**
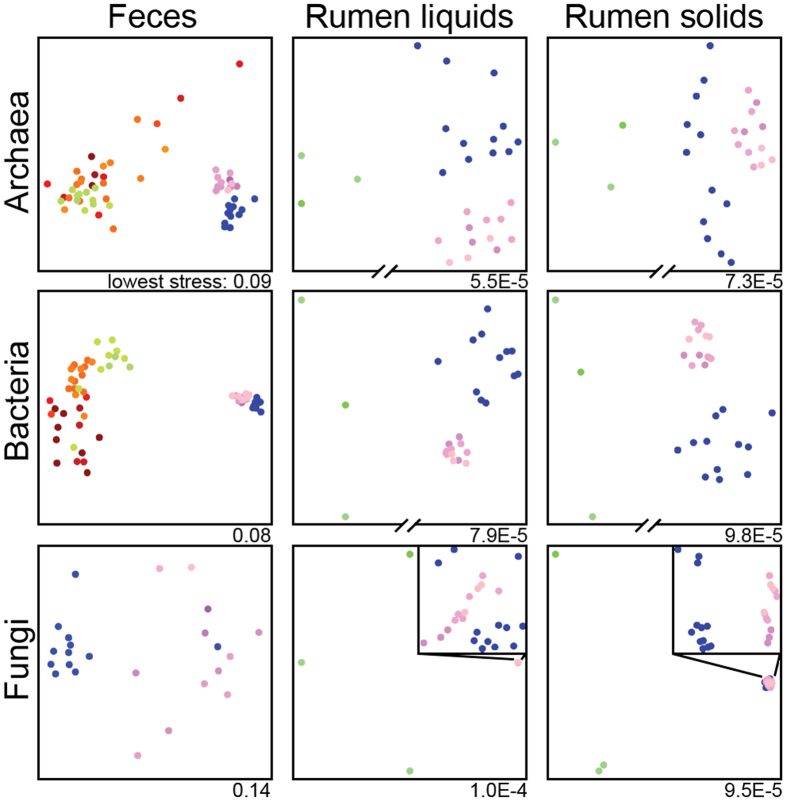
Non-metric multidimensional scaling (nMDS) plots of the Bray-Curtis diversity metric for microbial communities in the dairy cow GIT. Points are colored by animal age on a continuous rainbow scale with 2-week (red), 4-week (orange), 8-week (green), 1-year (blue), and 2-year (pink). Both archaeal and bacterial rumen plots have a split x-axis denoting a large change between 8 week and adult (1- and 2-year) samples. Fungal rumen plots have an inset plot displaying the 1- and 2-year cluster in more detail.

Age-related differences (2 weeks to lactation for feces, 8 weeks to lactation for rumen) were apparent in the archaeal, bacterial, and fungal communities of all sample types when visualized by nonmetric multidimensional scaling (nMDS) of the Bray-Curtis diversity metric (Fig. 1). Age groups were significantly different (ANOSIM, Supplementary Table 5) in all sample types for archaeal, bacterial, and fungal community structure (Bray-Curtis) and composition (Jaccard). Controls for animal identity among fecal communities and randomized groups among all communities were not significant for any amplicon.

**Figure 2:**
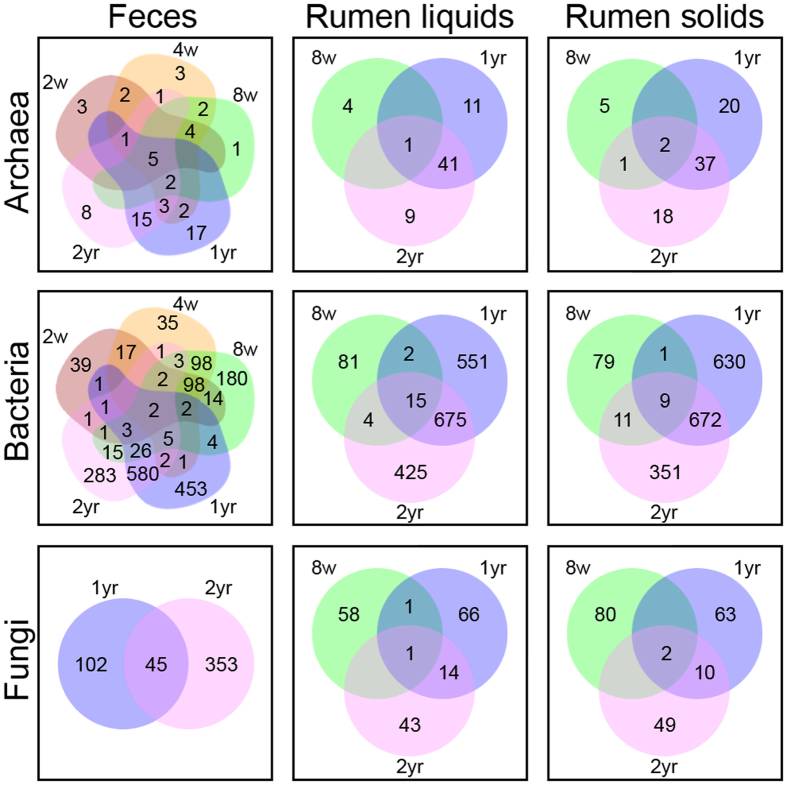
Shared OTUs among calves and adults at different ages according to sample type. Zeros are not shown, and only OTUs at a relative abundance of >1% in at least one sample were included in each group.

Inter-animal variation decreased with age, as evidenced by decreased spread of Bray-Curtis values within age groups (Fig. 1) as well as higher numbers of shared OTUs in older animals (Fig. 2). Both archaeal and bacterial communities in rumen liquids and solids showed decreased dispersion from 8 weeks to 1 year to 2 years (PERMDISP, *P* < 0.05), except archaeal dispersion in rumen solids which only showed a trend (PERMDISP *P* = 0.06, Supplementary Table 5). In feces, archaeal and bacterial communities were more dispersed at 2 and 4 weeks than at 8 weeks and continued to converge from 8 weeks to 1 year. In contrast, fungal communities had a significant change in dispersion from 1 to 2 years in rumen liquids. This apparent increased similarity among older individuals was caused by higher abundances of shared taxa, as opposed to loss of unshared taxa. This can be as seen by the positive linear relationship between the log mean change in abundance and log mean variance for individual calves in all pairwise age group comparisons (Supplementary Fig. 1), which indicates that more abundant OTUs had larger changes in relative abundance.

**Figure 3:**
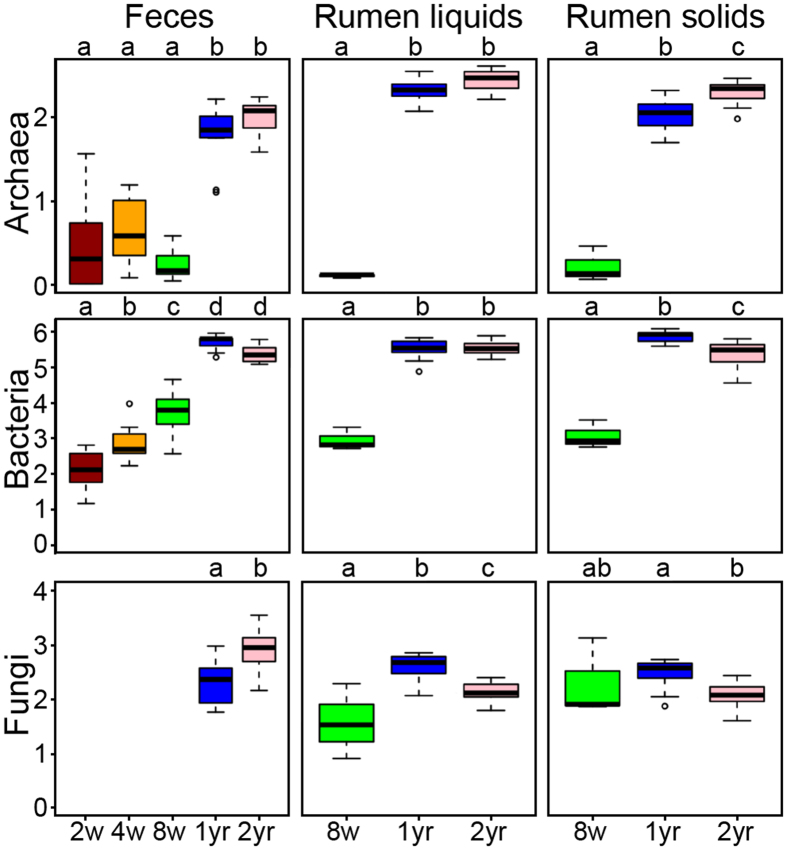
Shannon’s diversity index for microbial communities in the dairy cow GIT. Data are expressed as standard boxplots with medians. Outliers are shown as dots. Groups with different letters above the same plot are significantly different (Tukey’s HSD P < 0.05). Boxes are colored by animal age group: 2-week (red), 4-week (orange), 8-week (green), 1-year (blue), and 2-year (pink).

In general, community diversity (Shannon’s) and richness (Chao) changed as animals aged (2 weeks to lactation for feces, 8 weeks to lactation for rumen), ANOVA, Supplementary Table 5). Archaea and bacteria showed increased diversity in adult animals relative to calves for all sample types (Fig. 3, TukeyHSD, Supplementary Table 5). However, archaeal diversity in rumen solids increased from 1 to 2 years whereas bacterial diversity in rumen solids decreased. Rumen fungal communities increased in diversity from 8 weeks to 1 year but decreased from 1 to 2 years, while fecal fungal diversity increased from 1 to 2 years (Fig. 3). Similar trends were seen for community richness (Supplementary Fig. 2).

### Rumen OTUs, but few taxa, change with age in dairy cows

A number of archaeal, bacterial, and fungal OTUs in the rumen were found to correlate to animal age at 8 weeks, 1 year, or 2 years (Fig. 2, Table 1). A total of 12 rumen archaeal OTUs (A-OTUs) changed with age (SIMPER contribution >1%) and together, these A-OTUs accounted for 71–77% of the differences seen between age groups (cumulative SIMPER, Supplementary Table 6). In both rumen liquids and solids, *Methanobrevibacter* (A-OTU01) dominated at 8 weeks, and several different *Methanobrevibacter* (A-OTU02, 03, 05, 08) were highly abundant at 1 year or 2 years. In adult rumen liquids, several additional *Methanobrevibacter* (A-OTU06, 07, 09) were abundant, while in adult rumen solids, *Methanobrevibacter* (A-OTU11, 13) and vadinCA11 (A-OTU16, 25) were abundant.

In total, 31 rumen bacterial OTUs (B-OTUs) changed with age (SIMPER contribution >1%), accounting for 25–47% of the variation between age groups (cumulative SIMPER). In order to reach a cumulative SIMPER of 70% for comparisons in liquids or solids, 425 or 382 additional B-OTUs, respectively, with contributions as little as 0.04% were needed. Calf rumen liquids and solids varied between individuals but were characterized by several *Prevotella* (B-OTU023, 039, 057, 062, 068, 123, 217, 221) as well as *Acidaminococcus* (B-OTU079, 277), a *Succiniclasticum* (B-OTU080), and an unclassified Succinivibrionaceae (B-OTU002). Calf rumen liquids were further identified by a *Bulleidia* (B-OTU354), *Ruminococcus* (B-OTU089), and an unclassified p-2534-18B5 (B-OTU235), while calf rumen solids had a *Megasphaera* (B-OTU102), *Roseburia* (B-OTU275), and an unclassified Veillonellaceae (B-OTU104). Adult rumen bacterial communities contained different *Prevotella* (B-OTU004, 014, 019, 021, 022, 031), *Succiniclasticum* (B-OTU005, 027), and unclassified Succinivibrionaceae (B-OTU007) than calves. One-year rumen solids were further differentiated by unclassified Bacteroidales (B-OTU042, 114). Two-year rumen communities had *Prevotella* (B-OTU029, 070) and an unclassified Succinivibrionaceae (B-OTU002).

A total of 23 rumen fungal OTUs (F-OTUs) changed with age (SIMPER contribution >1%), accounting for 70–73% of the variation between age groups (cumulative SIMPER). Calf rumen liquid and solid communities differed between individuals with one to three dominating F-OTUs identified as *Caecomyces* (F-OTU007, 012, 066, 103, 360), *Piromyces* (F-OTU058), or unclassified fungi (F-OTU037, 046, 076, 202). Adult rumen samples contained highly abundant *Caecomyces* (F-OTU001, 002) and unclassified Neocallimastigaceae (F-OTU003, 006, 011). There were also several F-OTUs which were abundant in either 1-year (*Caecomyces* F-OTU007; *Orpinomyces* F-OTU008; *Piromyces* F-OTU005, 015; unclassified Neocallimastigaceae F-OTU010, 013, 014) or 2-year (*Caecomyces* F-OTU004; unclassified Neocallimastigaceae F-OTU019) liquid and/or solid rumen samples.

Total archaeal, bacterial, and fungal communities correlated in rumen liquids and solids (Mantel, *P* < 0.0001). In rumen liquids, 8-week samples had a number of co-occurring age-related OTUs (SIMPER). These included a *Methanobrevibacter* (A-OTU01) and an unclassified fungus (F-OTU046) with an *Acidaminococcus* (B-OTU079), a *Bulleidia* (B-OTU354), *Prevotella* (B-OTU023, 039, 217), and *Succiniclasticum* (B-OTU080, Kendall >0.70, Supplementary Fig. 3). A single 8-week rumen liquid sample was also characterized by a *Caecomyces* (F-OTU066) and *Piromyces* (F-OTU058) with *Prevotella* (B-OTU068, 123) and an unclassified p-2534-18B5 (B-OTU235). In contrast, 1- and 2-year rumen liquids contained less complex co-occurrence webs with only a *Methanobrevibacter* (A-OTU005), *Prevotella* (B-OTU070), and an unclassified Neocallimastigaceae (F-OTU019) co-occurring at 2 years.

Similar to liquids, 8-week rumen solids had a co-occurrence of age-related OTUs including a *Methanobrevibacter* (A-OTU01) and an unclassified fungus (F-OTU046) with an *Acidaminococcus* (B-OTU079) and *Prevotella* (B-OTU068, 123, 221) but also included *Caecomyces* (F-OTU066, 360), an unclassified fungus (F-OTU202), a *Megasphaera* (B-OTU102), and an unclassified Veillonellaceae (B-OTU104, Kendall >0.70, Supplementary Fig. 3). One-year rumen solids had associated *Methanobrevibacter* (A-OTU13), unclassified Bacteroidales (B-OTU484), vadinCA11 (A-OTU25), and *Succiniclasticum* (B-OTU027) with the fungi *Caecomyces* (F-OTU007), *Piromyces* (F-OTU005, 015, 016), and unclassified Neocallimastigaceae (F-OTU010, 013). Some 1-year liquids also had a *Methanobrevibacter* (A-OTU03) associated with an unclassified Bacteroides (B-OTU042). Two-year rumen solids tended to contain a vadinCA11 (A-OTU16), *Prevotella* (B-OTU22), and *Caecomyces* (F-OTU004). Those with *Caecomyces* (F-OTU004) also had other *Prevotella* (B-OTU021, 029) and an unclassified Succinivibrionaceae (B-OTU007). A subset of 2-year solids had a *Methanobrevibacter* (A-OTU05) with an unclassified Neocallimastigaceae (F-OTU019).

### Fecal taxa change with age in dairy cows

Overall, 7 fecal A-OTUs (Fig. 2, Table 1) changed with age from 2 weeks to over 2 years of age (SIMPER contribution >1%), and these A-OTUs explained 71–80% of age group differences in fecal samples (cumulative SIMPER, Supplementary Table 6). The same *Methanobrevibacter* (A-OTU01) found in rumen samples, as well as a *Methanosphaera* (A-OTU17), dominated fecal samples from 2 to 8 weeks. Different *Methanobrevibacter* OTUs (A-OTU02, 03, 05, 09) as well as an unclassified Methanocorpusculaceae (A-OTU04) had higher abundances in feces at 1 or 2 years (Supplementary Table 4).

Distinct from rumen bacterial communities, 36 different B-OTUs changed with age in feces (SIMPER contribution >1%), accounting for 18–69% of the observed age group differences (cumulative SIMPER). In order to reach a cumulative SIMPER of 70%, 228 additional B-OTUs with contributions as little as 0.07% were needed. B-OTUs important in calves included *Bacteroides* (B-OTU017, 018) and an unclassified S24-7 (B-OTU013), which all increased in abundance from 2 to 8 weeks but were not present in adults. Also, different *Bacteroides* (B-OTU009, 020) and an unclassified Enterobacteriaceae (B-OTU016) were variably abundant in calves but absent in adults. In contrast, many more B-OTUs tended to decrease in abundance as calves aged, reaching zero by 8 weeks and continuing to be absent in adult samples. These included a *Bifidobacterium* (B-OTU008), a *Blautia* (B-OTU035), a *Collinsella* (B-OTU006), a *Dorea* (B-OTU025), two *Faecalibacterium* (B-OTU001, 003), a *Lactobacillus* (B-OTU026), and two unclassified Ruminococcaceae (B-OTU015, 034). Adult fecal samples were characterized by a several unclassified Paraprevotellaceae (B-OTU038, 061, 091, 101) and Bacteroidaceae (B-OTU024, 032) as well as a *Bifidobacterium* (B-OTU046), *Treponema* (B-OTU028), an unclassified S24-7 (B-OTU043), and unclassified Bacteroidales (B-OTU067). These B-OTUs were absent in calf feces. Importantly, a *Succinivibrio* (B-OTU097) and an unclassified Ruminococcaceae (B-OTU012) were present in some calves and most adults. There were also a number of B-OTUs which were transiently associated with some calves at few time points (B-OTU010, 030, 064, 075, 106, 122, 152, 157, 170).

In total, 20 fecal F-OTUs changed with age (SIMPER contribution >1%) and together, accounted for 64% of the variation seen between 1- and 2-year fecal communities (cumulative SIMPER). In general, fecal fungal communities showed the same trends in the same F-OTUs as rumen communities. Notable exceptions included two *Caecomyces* (F-OTU001, 002) and an *Orpinomyces* (F-OTU003) which were present in 1-year rumen and fecal samples but did not persist in 2- year feces as they did in 2-year rumen samples. Also, several *Cyllamyces* (F-OTU009, 027), *Orpinomyces* (F-OTU018, 022 023), and unclassified fungi (F-OTU017, 039, 041, 044) were only appreciably abundant in 1- and/or 2-year feces.

Fecal communities had few strong correlations between specific A-, B-, and F-OTUs (Kendall >0.70, Supplementary Fig. 3), though total archaeal, bacterial, and fungal communities were found to correlate (Mantel, *P* < 0.0001). In 1-year feces, an unclassified Bacteroidaceae (B-OTU032) was associated with an unclassified Methanocorpusculaceae (A-OTU04), while 2-year feces had a *Methanobrevibacter* (A-OTU09) associated with a *Treponema* (B-OTU028) and an unclassified Bacteroidales (B-OTU067).

### Specific taxa correlate to animal growth and health

Animal growth, as defined by average daily gain (ADG) in weight (kg), correlated to the structure and/or composition of archaeal and fungal communities in 1-year rumen liquids while bacterial communities in 2-year rumen liquids correlated to ADG. Fungal communities in rumen solids also correlated to ADG at 1 year (PERMANOVA, Supplementary Table 5). One *Methanobrevibacter* in the rumen (A-OTU36) positively correlated to ADG (Kendall >0.70) while another *Methanobrevibacter* (A-OTU03) negatively correlated to ADG (Kendall <−0.70, Supplementary Table 6). Additionally, the rumen archaeon *vadinCA11* (A-OTU25) and a number of rumen fungi, including *Caecomyces* (F-OTU002), *Piromyces* (F-OTU015, 016), and an unclassified Neocallimastigaceae (F-OTU036), were positively correlated to ADG. A single bacterial OTU classified as *Prevotella* (B-OTU194) negatively correlated to ADG (Kendal <−0.70, Supplementary Table 6). Within the positively ADG-associated OTUs at 1-year, a vadinCA11 (A-OTU025) and *Piromyces* (F-OTU015) strongly co-occurred in rumen liquids, while two *Piromyces* (F-OTU015, 016) co-occurred in rumen solids.

Fecal bacterial community structure and composition were significantly different during scours (diarrhea, N = 5, ANOSIM, Supplementary Table 5). However, all scours samples were collected near 2 weeks, and within this age group, scours were not significant. Scours did not significantly impact archaeal communities and was not tested in fungal communities, as this amplicon was not sequenced for 2 week samples. Days with scours symptoms did not correlate to any microbial community in calves (PERMANOVA).

## Discussion

The gastrointestinal tract (GIT) microbiota plays important roles in host health and development, particularly in herbivores which require GIT microbes to break down their fiber-rich diet into accessible nutrients^2^. However, the complex process by which the ruminant GIT develops and acquires its microbiota is not fully understood. Here, we utilized next-generation sequencing to characterize the fecal microbiota of dairy cows from 2 weeks to the middle of the first lactation cycle and the rumen microbiota from 8 weeks to first lactation, as well as correlated these communities to animal growth and health.

Pre-weaning is a dynamic stage during which the calf must transition to extra-uterine life as well as gain weight and develop internally in preparation for weaning. While the calf can persist solely on milk, it is common practice to supplement their diet with calf starter grains during this stage to promote GIT development. This development includes the acquistion of specific GIT microbial communities, particularly those in the rumen, which are essential for survival on a plant-based diet post-weaning^2^. As pre-weaning calves are pre-ruminants, meaning they have an un- or under-developed rumen and much of their dietary intake by-passes the rumen, feces is a useful proxy for gut communities at this age and allows for sampling from animals over time.

We found that bacterial communities quickly establish in the calf GIT (feces) as early as 2 weeks of age, confirming previous reports^12^,^13^,^14^,^15^,^16^,^17^. In contrast, archaea were difficult to detect in feces at 2 and 4 weeks, and fungi were below detection until after weaning. Given that archaea and fungi were found to be established after bacterial colonization in the rumen of lambs^19^, we hypothesize that a similar succession occurs in cows, with the process further delayed in the distal gut. Under this model, bacteria quickly establish in the rumen and distal gut after birth to metabolize the milk and calf starter ingested by the calf. Once bacteria establish (2 to 4 weeks), methanogens can proliferate by using available bacterial-derived hydrogen to reduce carbon dioxide to methane^26^. Finally, colonization by fungi occurs once sufficient fiber from calf starter is present in the rumen (4 to 8 weeks) but are not fully established in the distal gut until dietary fiber is abundant, likely sometime after weaning.

Overall, the fecal microbial communities in pre-weaned calves were highly variable, with increasing alpha-diversity but decreasing inter-animal variation (beta-diversity) as the animals aged. This is consistent with previous reports of calf bacterial communities^11^,^12^,^13^,^14^,^15^,^16^, and our study further shows that this extends to archaea and fungi. Moreover, we found that convergence toward a similar microbiota is driven by increasing abundances of taxa shared between individuals, as opposed to decreases in unshared taxa. This supports a model where microbes from the environment are continually passsaged through the GIT until specific taxa can establish and proliferate, thus becoming part of the resident microbiota. Calves in this study had minimal maternal contact before being housed in individual hutches until after weaning, and their microbial exposure was likely limited to microorganisms present in the environment or from contact with farm workers. This may explain why the progression of taxa differed substantially between individuals with no strong co-occurences in OTUs in feces prior to weaning (8 weeks).

Pre-weaned calf fecal bacterial communities had high beta-diversity at the OTU-level but individuals were dominated by similar taxa including *Bacteroides*, Enterobacteriaceae, and S24-7. Bacteria in the genus *Bacteroides* and the family Enterobacteriaceae are commonly found in the feces of cows^11^ and human infants^27^, so their association with young calves is not surprising. Calf-associated OTUs within these taxa were not present in adult feces, indicating that the majority of the adult fecal microbiota does not begin to establish in animals prior to weaning. However, several calves also had low abundances of a *Succinivibrio* and a Ruminococcaceae OTU, which persisted in most adults. Both may be important in the adult fecal ecosystem as carbohydrate^28^ and fiber-degrading organisms^29^,^30^, and their presence in some calves suggests that early acquisition of specific adult-associated microorganisms can occur.

As the calf aged, many bacterial OTUs in feces decreased in abundance and disappeared by adulthood. These likely represent transient organisms or those associated with a primarily milk diet, which decreased in dominance in the GIT as calf starter intake increased with age. Most of these OTUs were saccharolytic genera including *Lactobacillus* and butyrate-producing *Faecalibacterium*^31^. Given that butyrate promotes ruminal and intestinal development in calves^32^, these bacteria likely aid in GIT development but are then out-competed by fiber-degraders in older animals. Similar trends have been seen previously for *Faecalibacterium*^11^,^12^,^16^ and *Lactobacillus*^11^,^12^ in calf feces, which highlights their general importance at this stage.

Once a dairy calf voluntarily consumes sufficient solid, supplemental feed, generally around 8 weeks of age, it is quickly transitioned off milk and calf starter grains to a more adult-like diet. While feces serve as a useful proxy of the overall GIT microbiota, weaning most strongly affects rumen communities since this is the site of the majority of fermentation of dietary fiber^2^. As a strictly anaerobic chamber, the rumen is a highly selective environment that must be colonized by particular microorganims if the animal is to survive post-weaning^2^. Thus, we investigated rumen communities in a limited number of animals in addition to fecal communities in all animals from 8 weeks onward. In general, microbial diversity increased in feces as calves were weaned (8 weeks) and rumen communities were present but not adult-like. Specifically, similar families and genera were present but few OTUs shared between animals at 8 weeks and 1 year, though it should be noted that rumen samples were obtained from different individuals at these time points. As expected, rumen bacteria were dominated by *Prevotella* with several different OTUs associated with different ages from 8 weeks to 2 years. *Prevotella* are abundant in the rumen and have diverse metabolic capabilities including fermentation of starches and xylan to yield propionate, succinate, and acetate^2^. Therefore, this taxon can fill a variety of niches, particularly during weaning when dietary substrates in the calf change substantially. This was also observed for OTUs in the family Succinivibrionaceae, which convert succinate to propionate that can be utilized by the host for gluconeogenesis to compensate for limited glucose availability^33^.

Similar to previous studies, calf rumen archaeal communities contained mostly methanogens within the genus *Methanobrevibacter*^34^. At weaning, a single *Methanobrevibacter* OTU dominated rumen and fecal communities, but this OTU was not present in adults where several other *Methanobrevibacter* OTUs dominated. Thus, it appears that the calf GIT provides a single methanogenic niche, likely defined by this taxa’s ability to use formate or hydrogen^35^,^36^ provided by co-occurring bacteria.

As with bacteria, calf fungal communities varied significantly among individuals of similar ages. In particular, the calf sampled a few days after weaning contained several fungal OTUs also present in adults and had a more diverse rumen community relative to the other two calves, which were sampled during weaning. This indicates that the calf fungal microbiota is quickly and significantly disrupted by the weaning transition. Since fungi mainly colonize fibrous solids^25^, we hypothesize that the sudden influx of new fibrous substrates after weaning allows previously low-abundant or transient fungi to persist and multiply. These organisms then compete for space and resources until an adult-like microbiota is reached sometime after weaning.

While the calf appears to acquire part of its adult microbiota during weaning, there were significant differences between weaned calves and adult cows. Thus, convergence toward a similar adult microbiota continues beyond weaning and important changes occur between weaning and 1 year. These include continued increases in GIT microbial diversity and dramatic decreases in beta-diversity from 8 weeks to 1 year. This is reflected in our OTU analysis of feces, which showed lower numbers of shared OTUs between weaning (8-week) and 1-year microbiotas, relative to calves (2 to 8 weeks). Once these animals transitioned to an adult microbiota, they converged toward a more similar microbiota, as evidenced by the large number of shared fecal and rumen taxa between 1- and 2-year old animals. This is likely due to microbial sharing between individuals, as calves were only co-housed after weaning. As seen in humans^37^, co-habitation facilitates transfer of microorganisms, allowing individuals to acquire a more similar, shared microbiota. This is further supported by studies in goats, where the rumen microbiota was similar in weaned and adult animals that were co-housed pre-weaning^38^.

Adult rumen communities were dominated by *Prevotella* and Succinivibrionaceae, and adult rumen and fecal fungal communities were dominated by cellulolytic and fibrolytic genera within the Neocallimastigaceae^39^, as has been seen previously^40^,^41^. However, these taxa were characterized by OTUs different than those in 8 week old calves, indicating continued species-level differences as animals age. Both adult rumen and fecal archaeal communities were dominated by *Methanobrevibacter* OTUs that were different from those in calves, confirming previous reports^26^,^42^. The adult archaeal microbiota was also more diverse than at weaning, likely as a result of the more diverse adult microbiota providing multiple substrates and methanogenic niches. This is supported by different *Methanobrevibacter* OTUs co-occurring with bacteria and fungi with diverse fermentative capabilities.

Interestingly, there were no clear associations between animal growth or health and specific OTUs in either fecal or rumen samples, and statistical power was limited for rumen samples from fewer animals. While overall rumen microbial communities correlated to weight gain by 1 or 2 years, OTUs associated with more or less weight gain were within the same genera, indicating species-level differences, or were within under-studied or unclassified genera, and therefore, difficult to assess. Of note, fungal OTUs were only found to positively correlate to weight gain, supporting their keystone role in the rumen digestive community^25^. Additional work in more individuals and in the rumen prior to weaning is necessary to more fully elucidate the role of the developing microbiota in calf health and growth.

In summary, our study is the first to track the fecal microbiota in a cohort of dairy calves from 2 weeks to first lactation as well as the rumen microbiota from weaning to first lactation. Our work shows that there is a succession of microbes within the calf GIT during development that converges upon an adult-like microbiota between weaning and 1 year of age. Given the interest in altering the cow microbiota for outcomes such as increased milk and meat production efficiency as well as reduced methane emissions, and the difficulty in altering the rumen microbiota of adult cows^24^, we suggest that such efforts may be most effective during the weaning transition. Manipulation of the GIT microbiota at this stage in life may result in the effective establishment of a target microbiota that remains stable into adulthood. Importantly, the observed microbial succession in our study may result from dairy calf management practices, as these animals experience minimal maternal care, develop in near isolation, and are weaned early relative to both beef cattle and what is considered “natural”^10^. While these practices are aimed at maximizing the milk available for sale, they likely force accelerated calf development, which may have long-term consequences on their GIT microbiota and downstream milk production.

## Materials and Methods

### Diet

A cohort of 15 Holstein dairy calves (3 male, 12 female) was raised at the US Dairy Forage Research Center farm (USDFRC, Prairie du Sac, WI). Calves were fed pasteurized milk with milk balancer protein-blend (Land O’Lakes, St. Paul, MN) added to 15% total milk solids. Animals were fed 5.8 L per day from 0 to 5 days and then 7.5 per day until weaning (8 weeks). Calves were also offered *ad libitum* calf starter supplement (58.25% whole corn, 1.75% molasses, and 40% Future Cow Ampli-Calf Mixer Pellet B150, Purina Animal Nutrition, Shoreview, MN). After weaning, calves were transitioned through a series of standard heifer diets until being placed on the lactating herd total mixed ration (TMR) diet after calving (Supplementary Table S1). All animals had *ad libitum* access to water throughout. All animal work was approved by the University of Wisconsin-Madison Institutional Animal Care and Use Committee under protocol A01501. All experiments were performed in accordance with the approved guidelines and regulations.

### Rumen collection

Samples were collected between June 2012 and February 2015. The three male calves were sacrificed at weaning (8 weeks) to obtain rumen samples. A subsample of total rumen contents was strained through four layers of cheesecloth to obtain rumen liquids. The remaining solids were squeezed to remove all liquid and transferred to a separate container. Four heifers were ruminally cannulated (9.5–11 months), and rumen solids and liquids were collected through the cannula before morning feeding on three consecutive days at 1 year (365 ± 7 days) and in the middle of first lactation cycle after 2 years (154–156 days in milk [DIM]). All samples were immediately transported on wet ice and stored at −80 °C prior to DNA extraction.

### Fecal collection

Fresh feces were obtained by-hand from the rectum of animals using clean gloves. Samples were taken at 2 weeks (13 ± 2 days), 4 weeks (27 ± 2 days), 8 weeks (54 ± 2 days), and 1 year (364 ± 6 days) of age as well as after 2 years during the middle of the first lactation cycle (155 ± 1 DIM). Samples were stored at −20 °C on site, transported on wet ice, and then stored at −80 °C prior to DNA extraction.

### Growth and health

Successful immunological passive transfer was tested by refractometer analysis of blood serum total protein (BSTP); only calves with a BSTP greater than 5 were enrolled in the study. Calf health was assessed by standard monitoring for disease including daily fecal (firm [1] to watery [4]) and attitude scoring (alert [1] to unresponsive [4]). A calf was considered to have scours with a fecal score of 3 and severe scours with a score of 4; fecal samples were not taken during severe scours. Calves were treated with antibiotics and electrolytes for scours (diarrhea) and with antibiotics plus a fever reducer for respiratory disease. Calves were weighed at 2 days and near the time of fecal sampling from 2 to 8 weeks. Cows were weighed on three consecutive days at 1 year (365–367 days) and 2 years (149–151 DIM).

### DNA extraction

Total genomic DNA was extracted following a mechanical disruption and phenol extraction protocol^43^ with feces treated as rumen solids and with the following modification: 25:24:1 phenol:chloroform:isoamyl alcohol was used in place of phenol:chloroform and samples required up to six additional washes. All DNA samples were resuspended in water, quantified by Qubit^®^ Fluorometer (Invitrogen, San Diego, CA, USA), and stored at −20 °C.

### Bacterial amplification and sequencing

PCR was performed using universal primers flanking the variable 4 (V4) region of the bacterial 16 S rRNA gene^44^. A total of 50 ng DNA, 0.4 μM each primer, 12.5 μl 2X HotStart ReadyMix (KAPA Biosystems, Wilmington, MA, USA), and water to 25 μl were used for one reaction per sample. Cycling conditions were as follows: initial denaturation of 95 °C for 3 min followed by 25 cycles of 95 °C for 30 s, 55 °C for 30 s, and 72 °C for 30 s, with a final extension at 72 °C for 5 min. PCR products were purified by gel extraction from a 1.0% low-melt agarose gel (National Diagnostics, Atlanta, GA) using a ZR-96 Zymoclean Gel DNA Recovery Kit (Zymo Research, Irvine, CA). Samples were quantified by Qubit^®^ Fluorometer and equimolar pooled. The pool plus 5% PhiX control DNA was sequenced with the MiSeq 2 × 250 v2 kit (Illumina, San Diego, CA, USA) using custom sequencing primers^44^.

### Archaeal and fungal amplification and sequencing

A two-step PCR protocol was employed for archaeal and fungal amplicons. The first PCR used universal primers flanking the V6-8 16 S region for archaea or the internal transcribed region 1 (ITS1) for fungi^40^. PCR reactions and cycling conditions were as previously described except fungi were amplified for 35 cycles. Products were column purified using the PureLink Pro 96 PCR Purification kit (Invitrogen). The second PCR was performed using 5 μl of cleaned PCR product with primers to add Illumina adapters and unique indices for each sample. Reactions and conditions were as previously described except 8 cycles were completed. Products were gel purified, quantified, and pooled as before. The pool along with 5% PhiX control DNA was sequenced with the MiSeq 2 × 300 v2 kit (Illumina). All DNA sequences have been deposited in the NCBI Short Read Archive (SRP073728).

### Sequence clean-up

All sequences were demultiplexed on the Illumina MiSeq. Further sequence processing was performed using mothur v.1.36.1^45^ following an adapted protocol^44^ (Supplementary Text S1). Briefly, paired-end sequences were combined into contigs and poor quality sequences were removed. Bacterial and archaeal sequences were aligned against the SILVA 16 S rRNA gene reference alignment database^46^ and screened for alignment to the correct region. Due to the frequency of insertions and deletions in ITS, fungal sequences were not aligned to a database^47^, and a de novo alignment within the dataset was performed instead. For all amplicons, sequences were pre-clustered to reduce sequencing error and chimera detection and removal was performed. Bacterial and archaeal sequences were classified to the GreenGenes database^48^; fungal sequences were classified to the UNITE dynamic ITS database^49^. Singletons were removed to facilitate downstream analyses.

### Sequence analysis and statistics

All sequences were grouped into 97% operational taxonomic units (OTUs) by uncorrected pairwise distances and furthest neighbor clustering. Coverage was assessed by Good’s coverage calculated in mothur. Bacterial (9000 seqs), archaeal (375), and fungal (250) communities were normalized to equal sequence counts, and these normalized OTU tables were used in all further analyses. Alpha-diversity was assessed with Shannon’s diversity and Chao’s richness calculated in mothur. Differences in community diversity and richness were assessed overall by analysis of variance (ANOVA) with the Benjamini-Hochberg correction for multiple comparisons and pairwise between age groups by Tukey’s honest significant difference (HSD) from multiple comparisons in R v3.2.3^50^.

Beta-diversity was visualized by Venn diagrams^51^ of OTUs at >0.1% relative abundance, and nonmetric multidimensional scaling (nMDS) plots of the Bray-Curtis metric calculated with square root transformed data in R (vegan package)^52^. Differences in the spread of Bray-Curtis values within groups were calculated by permutation tests of multivariate homogeneity of group dispersions (PERMDISP) in vegan with the Benjamini-Hochberg correction for multiple comparisons. Trends causing changes in dispersion were identified by log-linear models. Changes in log OTU abundances were compared by animal, pairwise between age groups, and corrected for number of days between samples. Only OTUs present in at least two samples in each pairwise comparison were included. Changes in OTU relative abundances between age groups were assessed by linear models of log mean abundance by log mean variance to indicate if more or less abundant OTUs had higher or lower changes in relative abundance with animal age.

Total community structure (relative abundance, Bray-Curtis) and composition (presence/absence, Jaccard) were evaluated for changes using analysis of similarity (ANOSIM, vegan) with the Benjamini-Hochberg correction for multiple comparisons. Communities were assessed by age groups, scours (diarrhea) and as controls, animal identity and random groups. OTUs contributing to age group differences seen in ANOSIM were identified by analysis of similarity percentages (SIMPER) in vegan. OTUs contributing at least 1% of the variation up to a cumulative total of 70% in at least one pairwise comparison were considered significant. Correlations between total archaeal, bacterial, and fungal communities were assessed by Mantel tests for dissimilarity matrices in R (vegan). Specific correlations between OTUs within significant Mantel tests were measured by Kendall’s rank correlation.

Total community structure and composition were also assessed for changes in relation to animal growth and health. Growth was measured by average daily gain (ADG) in weight (kg) relative to 2 day measurements. Health was measured by cumulative days with scours (diarrhea) or respiratory disease in calves or any disease symptoms in adults. Communities were assessed by permutational ANOVA (PERMANOVA, vegan) within age groups independently and with the Benjamini-Hochberg correction for multiple comparisons, because variables differed with age and were continuous, thus not suitable for use in ANOSIM. To avoid animal effects in adult rumen samples, PERMANOVA tests were performed on OTU tables averaged across the 3 consecutive sampling days. Correlations of ADG or days ill to specific OTUs were measured by Kendall’s rank correlation of OTUs at a minimum of 0.5% abundance in at least one sample. All tests were assessed at significance *P* < 0.05, and strong correlations were defined as either >0.7 or <−0.7. R code may be found in Text S1.

## Additional Information

**How to cite this article**: Dill-McFarland, K. A. *et al*. Microbial succession in the gastrointestinal tract of dairy cows from 2 weeks to first lactation. *Sci. Rep.* **7**, 40864; doi: 10.1038/srep40864 (2017).

**Publisher's note:** Springer Nature remains neutral with regard to jurisdictional claims in published maps and institutional affiliations.

## Supplementary Material

Supplementary Information

Supplementary Table S4

## Figures and Tables

**Table 1:** Age-associated OTUs within the dairy cow GIT microbiota.

Kingdom	Sample	Family (order if unclassified)	Genus	Pre-weaning	Weaning	Adult
Archaea	RF	Methanobacteriaceae	*Methanobrevibacter*	■●●●	■	○○○○○○●●●
		Methanobacteriaceae	*Methanosphaera*	■	■	
		Methanocorpusculaceae	unclassified			○○
		Methanomassiliicoccaceae	vadinCA11			○○
Bacteria	R	Erysipelotrichaceae	*Bulleidia*	NA	○	
		Lachnospiraceae	*Roseburia*	NA	○	
		p-2534-18B5 (order Bacteroidales)	unclassified	NA	○	
		Paraprevotellaceae	*Prevotella*	NA	○	
		Prevotellaceae	*Prevotella*	NA	○○○○○○○○	○○○○○○○○
		Ruminococcaceae	*Ruminococcus*	NA	○	
		Succinivibrionaceae	unclassified	NA	●	○●
		Veillonellaceae	*Acidaminococcus*	NA	○○	
		Veillonellaceae	*Megasphaera*	NA	○	
		Veillonellaceae	*Succiniclasticum*	NA	○	○○
		Veillonellaceae	unclassified	NA	○	
		unclassified (order Bacteroidales)	unclassified	NA		○○○
	F	Bacteroidaceae	*Bacteroides*	○■■■■	■■■■	
		Bacteroidaceae	unclassified			○○
		Bifidobacteriaceae	*Bifidobacterium*	○●		●
		Clostridiaceae	*Clostridium*	■	■	
		Coriobacteriaceae	*Collinsella*	■	■	
		Coriobacteriaceae	unclassified		○	
		Enterobacteriaceae	unclassified	■	■	
		Lachnospiraceae	*Blautia*	○○■	■	
		Lachnospiraceae	*Dorea*	■	■	
		Lactobacillaceae	*Lactobacillus*	○		
		Paraprevotellaceae	CF231			○○○○
		Peptostreptococcaceae	*Peptostreptococcus*	○		
		Prevotellaceae	unclassified	○		
		S24-7 (order Bacteroidales)	unclassified	■	■	○
		Spirochaetaceae	*Treponema*		○	○
		Succinivibrionaceae	*Succinivibrio*		●	●
		Ruminococcaceae	*Faecalibacterium*	■■	■■	
		Ruminococcaceae	unclassified	■■	■■●	●
Fungi	RF	Neocallimastigaceae	*Caecomyces*		○●●	●●○○○
		Neocallimastigaceae	*Cyllamyces*			○○
		Neocallimastigaceae	*Orpinomyces*			○○○○
		Neocallimastigaceae	*Piromyces*		○○○	○○
		Neocallimastigaceae	unclassified			○
		unclassified fungi	unclassified		○○○○	○○○○○○○○○○

OTUs which changed significantly between at least one pairwise age group comparison (SIMPER contribution >1%). Archaeal and fungal OTUs are grouped for rumen and fecal samples (RF) due to significant similarities between sample types. OTUs found in only one group are identified by open circles; OTUs found in both calf groups (2, 4, and 8 weeks) are indicated by closed squares; and OTUs in adults and only one calf group (2, 4 weeks or 8 weeks) by closed circle.
