# Supplementary material for: Microbial succession in the gastrointestinal tract of dairy cows from 2 weeks to first lactation
Source: Sci Rep. 2017 Jan 18;7:40864. doi: 10.1038/srep40864 (PMC5241668; doi:10.1038/srep40864)
Supplement: Supplementary Information [file srep40864-s1.pdf]

Microbial succession in the gastrointestinal tract of dairy cows from 2 weeks to first lactation

Kimberly A. Dill-McFarland,[a] Jacob D. Breaker,[a] Garret Suen[a]*

Department of Bacteriology, University of Wisconsin-Madison, Madison, Wisconsin, USA[a]

Running Head: Microbial succession in the gut of dairy cows

*Address correspondence to Garret Suen, gsuen@wisc.edu

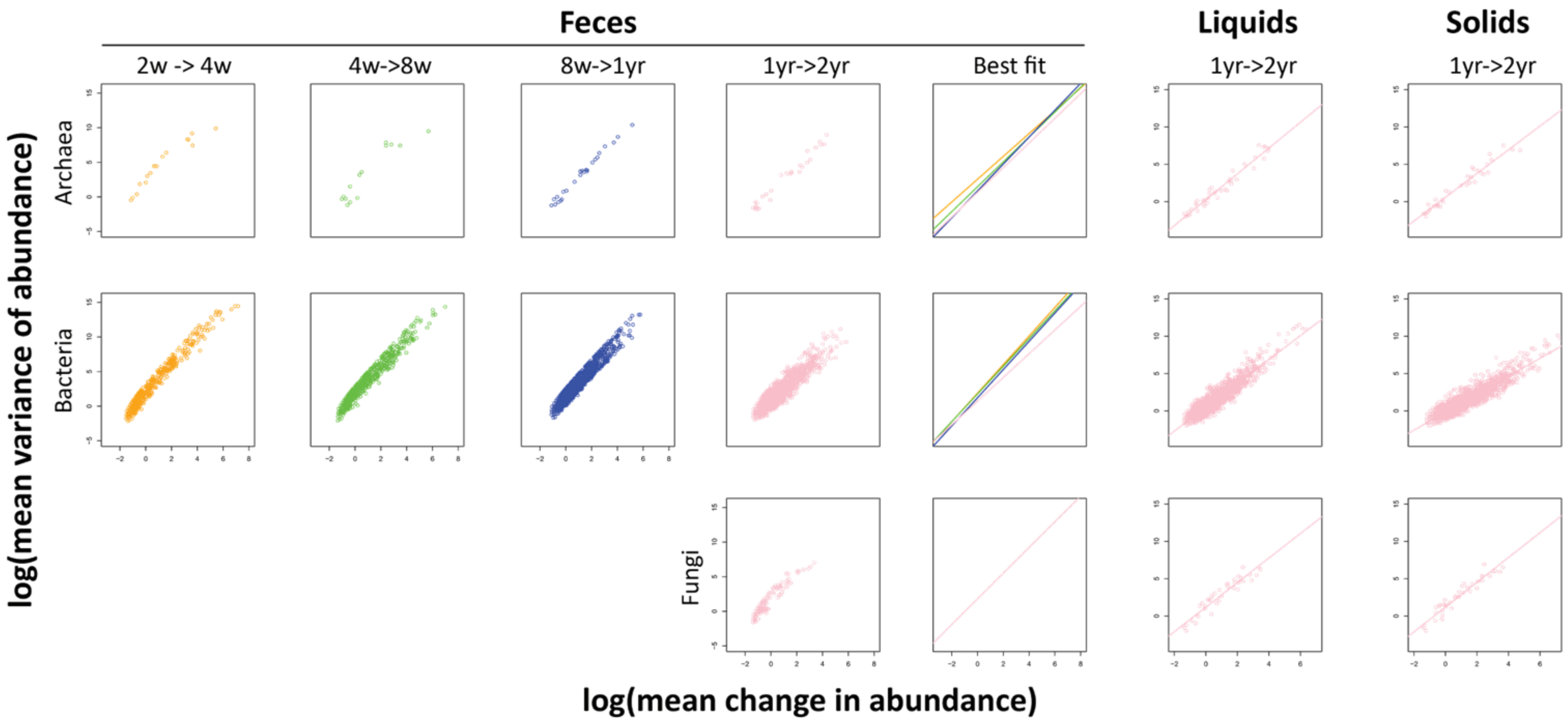

Fig. S1. Mean change in abundance versus mean variance of abundance of OTUs. Log OTU abundances were compared by animal, pairwise between age groups, and corrected for number of days elapsed between samplings. Only animals with both samples in the pairwise comparison and OTUs present in at least two samples were included. Rumen liquid and solid 8 week samples were not compared to 1-year samples, because they came from different animals.

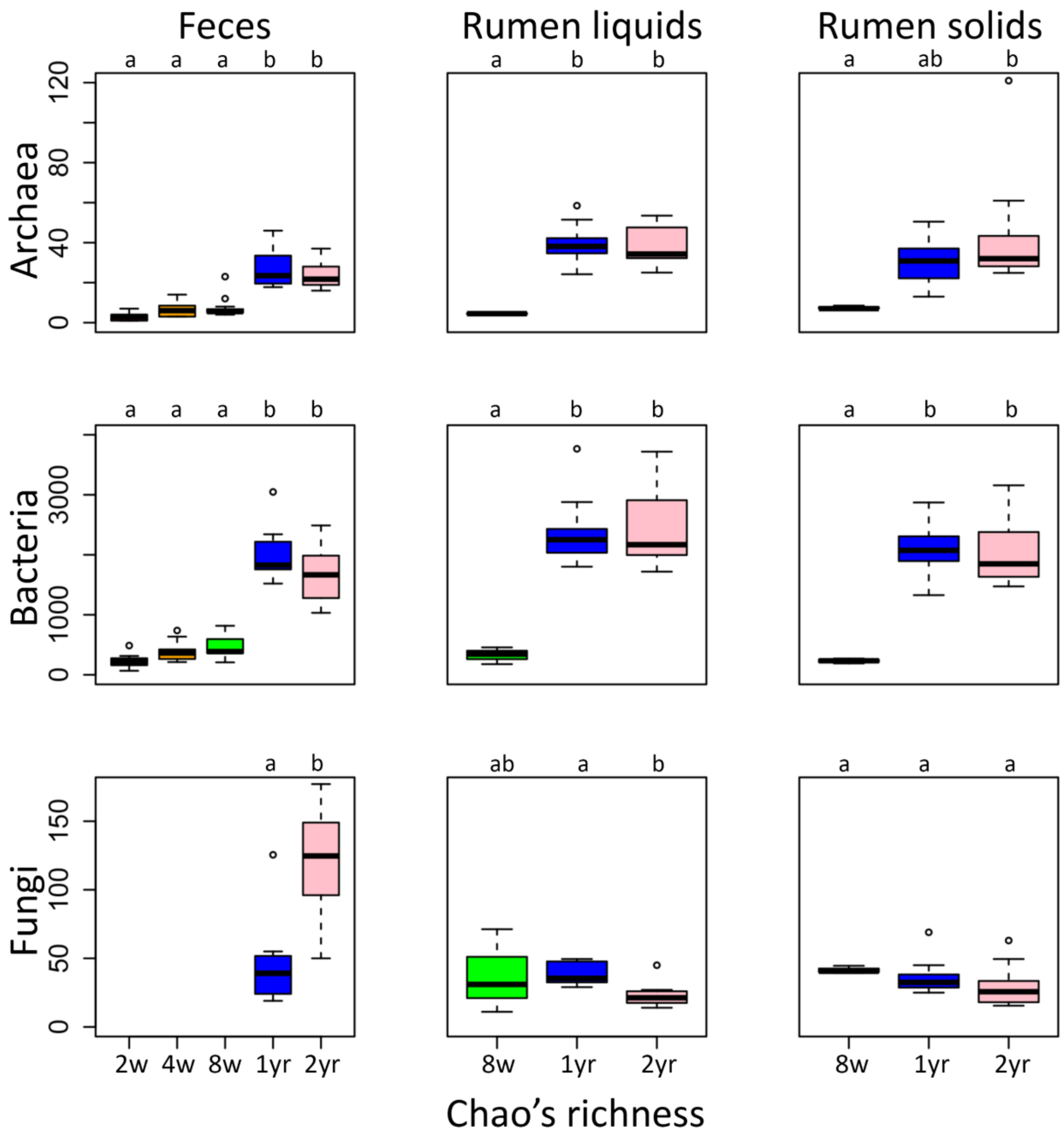

Fig. S2. Chao's richness estimate for archaeal, bacterial, and fungal communities in dairy cow feces as well as rumen liquids and solids. Data are expressed as boxplots with outliers shown as dots. Groups with different letters within the same plot are significantly different (Tukey's HSD $P < 0.05$). Boxes are colored by animal age group: 2-week (red), 4-week (orange), 8-week (green), 1-year (blue), and 2-year (pink).

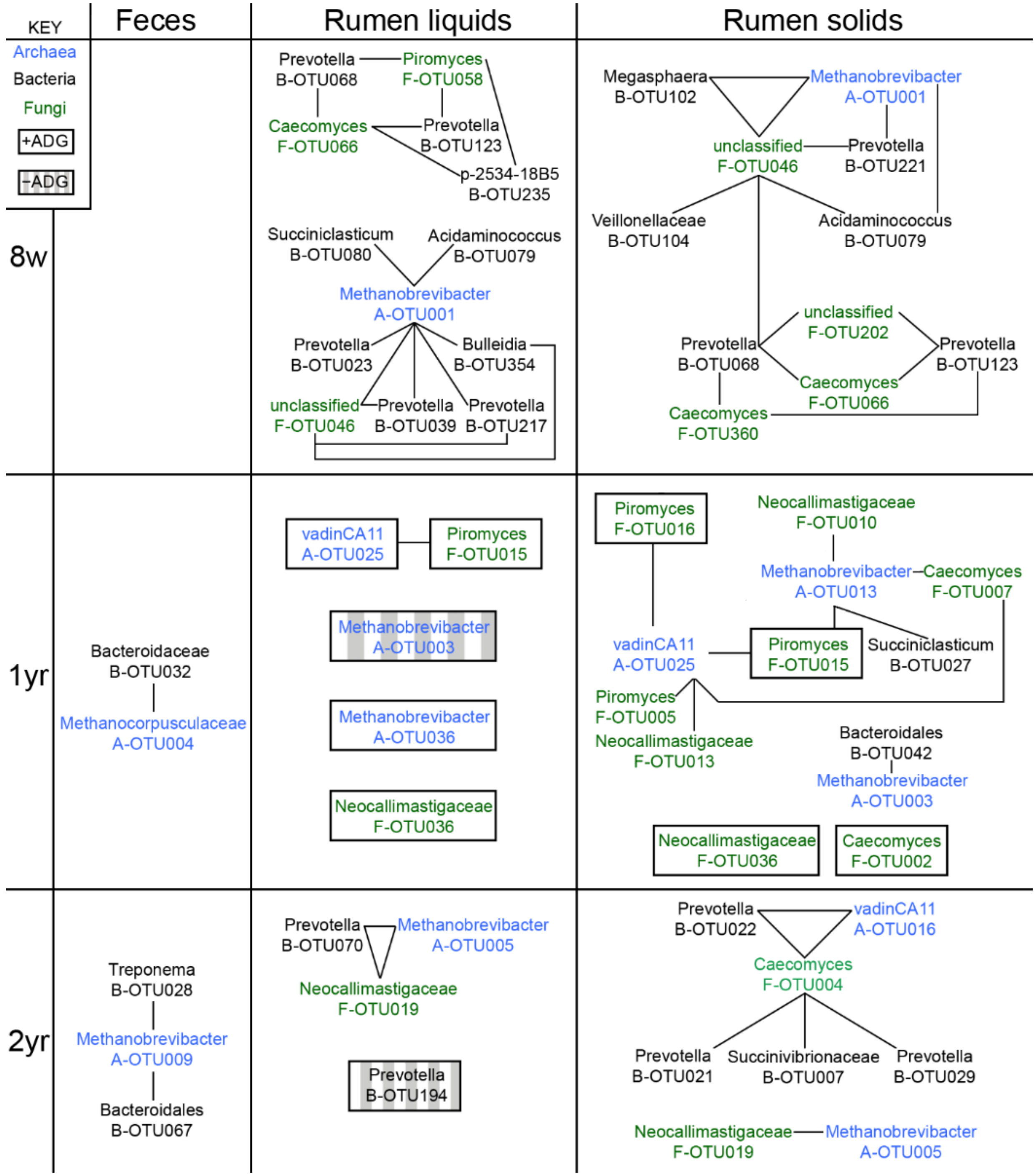

Fig. S3. Co-occurrence networks of OTUs significantly different by animal age and/or growth. OTUs at a relative abundance of 0.5% or greater in at least one sample, found to change with age (SIMPER), and co-occur (Kendall) are shown unboxed. OTUs above the abundance cutoff and also found to correlate to animal growth (ADG, Kendall) are shown in boxes. Open shapes indicate a positive correlation (Kendall > 0.70), while lines indicate a negative correlation (Kendall < -0.70). Solid lines between OTUs indicate strong co-occurrence (Kendall > 0.70). Archaea are blue, bacteria are black, and fungi are green.

Table S1a. Nutritional analysis of feed components by % dry matter (DM)

	CP	Starch	Sugar (ESC)	Fat (EE)	Ash	ADF	NDF	Ca	Mg	P	K	S	Lasalocid (antibiotic, g/ton)
Calf starter	22.68	42.30	1.75	2.82	6.52	5.18	11.06	1.02	0.22	0.59	1.13	0.28	60
Hay silage	22.5	3.1	-	2.8	-	30.1	37.5	1.3	-	0.40	2.83	-	0
Corn silage	7.2	33.3	-	3.4	-	23.9	39	0.23	-	0.23	0.7	-	0
Cracked corn	8.1	73.7	-	4.3	-	3.5	9.7	0.02	-	0.29	0.44	-	0
Heifer base rum	0	0.01	-	0	-	0	0	25.4	-	1.13	1.14	-	0
Grass silage base	14.5	3.6	-	3	-	37	52	0.83	-	0.36	2.9	-	0
Wheat straw	3.8	-	-	1.7	-	54.3	80.4	0.27	-	0.08	1.4	-	0
Heifer base	0	0.008	-	0	-	0	0	25.9	-	1.11	1.13	-	0
Corn, high moisture shell	8.2	69.4	-	2.9	-	2.6	7.0	0.03	-	0.33	0.44	-	0
Corn, grade shell	9.0	68.0	-	4.2	-	3.5	9.7	0.03	-	0.29	0.44	-	0

Values are averages across the sampling period as crops vary slightly by year. CP: crude protein, ESC: ethanol soluble carbohydrates, EE: ether extraction, ADF: acid detergent fiber, NDF: neutral detergent fiber

Table S1b. Feed components offered at different ages (%DM)

	0-8 weeks	2-3 months*	3-5 months	6-10 months	11-14 months	15+ months	Lactating
Calf starter	100	17.3 - 100	17.3	0	0	0	0
Hay silage	0	0 - 44.5	44.5	32.1	38.6	39.0	28.8
Corn silage	0	0 - 22.2	22.2	30.7	25.7	20.6	28.8
Cracked corn	0	0 - 14.8	14.8	8.9	0	0	0
Heifer base rum	0	0 - 1.2	1.2	1.5	1.4	0	0
Grass silage base	0	0	0	16.1	17.2	21.7	0
Wheat straw	0	0	0	0	14.3	17.4	0
Heifer base	0	0	0	0	0	1.3	0
Dry distillers grains	0	0	0	10.7	2.9	0	4.2
Corn, high moisture shell	0	0	0	0	0	0	16.3
Corn, grade shell	0	0	0	0	0	0	5.5
Soybeans	0	0	0	0	0	0	7.4
Canola meal	0	0	0	0	0	0	6.5
USDA minerals	0	0	0	0	0	0	2.5

*This time period was transitional with a gradual daily change from the 8 week to the 3 month diet

Table S2. Metadata on calf sex, age, growth, and health.

			Age (dy)			Growth	Health (cumulative dy)					
Calf	Age group	Sex	Fecal sampling	Rumen sampling	Weaning	ADG (kg/dy)	Scour sample	Scours	Resp	Ketosis	Mastitis	All
4924	2w	M	22			1.54	N	0	0	0	0	0
4924	4w	M	29			1.16	N	0	0	0	0	0
4924	8w	M	54	58	58	1.59	N	0	0	0	0	0
4956	2w	M	12			1.45	N	0	0	0	0	0
4956	4w	M	25			0.89	N	0	0	0	0	0
4956	8w	M	54	55	55	1.36	N	0	0	0	0	0
4971	2w	M	12			0.36	Y	8	0	0	0	8
4971	4w	M	28			1.31	N	10	0	0	0	10
4971	8w	M	56	62	60	1.23	N	10	0	0	0	10
5017	2w	F	13			1.00	N	0	0	0	0	0
5017	4w	F	26			0.60	N	9	0	0	0	9
5017	8w	F	53		55	1.38	N	9	0	0	0	9
5017	1yr	F	369	369-371		0.88	N	9	0	0	0	9
5017	2yr	F	930	929-931		0.52	N	9	0	0	0	9
5020	2w	F	15			1.01	N	0	0	0	0	0
5020	4w	F	27			1.03	N	0	0	0	0	0
5020	8w	F	54		53	1.00	N	0	0	0	0	0
5020	1yr	F	365			1.07	N	0	0	0	0	0
5020	2yr	F	895			0.70	N	0	0	0	0	0
5026	2w	F	11			1.56	N	0	0	0	0	0
5026	4w	F	26			1.10	N	1	0	0	0	1
5026	8w	F	53		59	1.23	N	1	0	0	0	3
5026	1yr	F	365			0.98	N	1	0	0	0	8
5026	2yr	F	803			0.67	N	1	0	1	0	9

5031	2w	F	15			1.22	Y	6	0	0	0	6
5031	4w	F	27			0.71	N	11	0	0	0	11
5031	8w	F	53		56	1.37	N	11	0	0	0	11
5031	1yr	F	368	368-370		0.86	N	14	0	0	0	14
5031	2yr	F	843	842-844		0.60	N	14	0	0	9	23
5037	2w	F	11			0.35	N	5	0	0	0	5
5037	4w	F	25			0.18	N	11	6	0	0	17
5037	8w	F	54		58	0.95	N	15	6	0	0	21
5037	1yr	F	358			0.94	N	18	6	0	0	24
5037	2yr	F	841			0.63	N	18	6	0	0	24
5041	2w	F	11			1.11	Y	5	0	0	0	5
5041	4w	F	25			1.89	N	9	0	0	0	9
5041	8w	F	54		52	1.35	N	9	0	0	0	9
5041	1yr	F	364			0.93	N	9	0	0	0	17
5041	2yr	F	843			0.72	N	9	0	0	0	17
5045	2w	F	14			0.91	N	4	6	0	0	10
5045	4w	F	25			1.83	N	4	6	0	0	10
5045	8w	F	55		52	0.90	N	4	6	0	0	10
5045	1yr	F	367	367-369		0.91	N	4	6	0	0	18
5045	2yr	F	868	867-869		0.72	N	4	6	0	0	18
5046	2w	F	14			1.26	N	0	5	0	0	5
5046	4w	F	25			1.77	N	0	5	0	0	5
5046	8w	F	NS		58							
5046	1yr	F	367			0.85	N	0	5	0	0	24
5046	2yr	F	867			0.61	N	0	5	0	0	24
5049	2w	F	11			0.76	N	0	6	0	0	6
5049	4w	F	26			0.64	N	0	6	0	0	6
5049	8w	F	NS		59							
5049	1yr	F	359			0.86	N	0	6	0	0	26

5049	2yr	F	867			0.66	N	0	6	0	9	35
5053	2w	F	11			0.05	Y	2	0	0	0	2
5053	4w	F	27			1.42	N	6	0	0	0	6
5053	8w	F	55		53	0.88	N	18	12	0	0	30
5053	1yr	F	365	365-367		0.93	N	18	12	0	0	38
5053	2yr	F	827	826-828		0.60	N	18	12	0	0	44
5058	2w	F	11			0.76	Y	1	0	0	0	1
5058	4w	F	25			1.10	N	5	0	0	0	5
5058	8w	F	NS		54							
5058	1yr	F	364			0.94	N	5	0	0	0	14
5058	2yr	F	752			0.75	N	5	0	0	0	14
5063	2w	F	14			0.49	N	0	0	0	0	0
5063	4w	F	28			1.01	N	5	0	0	0	5
5063	8w	F	NS		54							
5063	1yr	F	360			0.86	N	5	0	0	0	12
5063	2yr	F	862			0.66	N	5	0	1	9	22

NS: no sample taken when one was planned.
ADG: Average daily gain

Table S3. Sequence coverage.

			ARCHAEA							BACTERIA							FUNGI						
Calf	Age	Type	Raw	Clean	Abund	OTUs	Coverage	Norm	Norm OTUs	Raw	Clean	Abund	OTUs	Coverage	Norm	Norm OTUs	Raw	Clean	Abund	OTUs	Coverage	Norm	Norm OTUs
4924	2w	F	38753	406	366	7	100.0%	375	7	25092	19137	18994	211	99.7%	8971	159							
4924	4w	F	37175	813	730	11	99.7%	377	11	54693	44664	44472	257	99.9%	8970	143							
4924	8w	F	30399	5006	4595	11	100.0%	373	4	26262	17632	17538	281	99.7%	9040	281							
4924	8w	L	27667	4877	4554	7	100.0%	375	4	66983	49926	49771	243	99.9%	8988	164	8369	2193	1438	34	99.7%	247	19
4924	8w	S	34793	1440	1306	9	99.8%	375	6	56733	46007	45836	235	99.9%	8985	136	29376	11002	7430	96	99.9%	244	21
4956	2w	F	37485	21	15	3	93.3%	375	3	27150	12715	12616	73	99.9%	8998	73							
4956	4w	F	18227	216	182	3	99.5%	375	3	35626	27991	27830	265	99.8%	9009	222							
4956	8w	F	24558	5748	5343	7	100.0%	374	3	98423	68898	68562	655	99.9%	8965	406							
4956	8w	L	20795	4561	4184	8	100.0%	375	4	94976	58398	58206	254	99.9%	8969	130	32729	12604	10589	42	100.0%	248	10
4956	8w	S	22610	2823	2576	8	100.0%	373	4	72470	44647	44479	215	99.9%	8982	144	33977	21169	17151	152	100.0%	241	25
4971	2w	F	36883	1	0	0	0.0%	0	0	78382	37751	37591	191	99.9%	8965	105							
4971	4w	F	26135	750	675	5	99.4%	377	5	131637	106001	105723	488	99.9%	8966	222							
4971	8w	F	14099	1844	1714	8	99.9%	374	5	83489	56910	56635	623	99.9%	8938	408							
4971	8w	L	32448	5619	5236	8	100.0%	374	4	102314	49188	48893	328	99.9%	8980	201	18919	5908	4373	69	99.9%	247	33
4971	8w	S	17192	537	486	7	99.4%	377	7	105803	55523	55285	349	99.9%	8947	170	12491	3830	2820	91	99.9%	240	42
5017	2w	F	30989	4916	4577	9	100.0%	374	4	78629	53505	53322	290	99.9%	9002	186							
5017	4w	F	24383	1092	979	8	99.7%	376	5	40857	33419	33244	301	99.8%	9007	242							
5017	8w	F	43652	11397	10786	7	100.0%	375	4	42976	30682	30347	453	99.7%	9010	368							
5017	1yr	F	15263	2562	2284	30	99.8%	372	18	68486	41465	40743	1834	99.5%	8841	1160	86447	53219	40737	375	99.9%	238	18
5017	1yr	L1	36150	2637	2335	43	99.7%	370	25	91567	55906	55197	2662	99.2%	8694	1439	70063	37070	29489	405	99.9%	237	27
5017	1yr	L2	28270	3120	2727	40	99.8%	374	26	98193	63991	63177	2578	99.3%	8805	1386	38419	17704	13656	261	99.7%	238	27
5017	1yr	L3	19761	2020	1757	34	99.9%	370	22	24097	15320	15012	1394	98.0%	9066	1394	66123	26394	19963	376	99.8%	236	28
5017	1yr	S1	16769	680	591	25	99.3%	376	25	63086	37577	37086	2174	99.0%	8711	1339	72073	24044	18185	346	99.8%	235	26
5017	1yr	S2	20235	518	442	21	99.5%	378	21	16038	9899	9637	1188	97.0%	9137	1188	70719	29207	22063	398	99.8%	236	27
5017	1yr	S3	23609	1129	933	33	99.2%	372	26	79659	54209	53440	2475	99.2%	9026	1661	84079	35218	28482	342	99.9%	238	26
5017	2yr	F	18382	1431	1253	23	99.7%	374	19	37498	24599	24262	1265	99.1%	8941	1039	68670	34791	25149	740	99.9%	202	59
5017	2yr	L1	24718	4178	3619	54	99.9%	372	29	54153	31637	31140	1802	98.9%	9026	1474	32393	18220	13605	183	99.8%	239	14
5017	2yr	L2	21520	2797	2435	48	99.6%	369	23	48154	29695	29274	1741	98.9%	9049	1407	37549	14618	10474	185	99.8%	238	16
5017	2yr	L3	32408	3220	2808	44	99.8%	373	28	67361	43069	42345	2044	99.1%	8817	1285	69234	28634	20197	348	99.9%	236	17
5017	2yr	S1	20759	942	835	27	99.8%	375	25	62079	41263	40573	1787	99.2%	8838	1114	46251	27966	21387	248	99.9%	239	13
5017	2yr	S2	13379	452	396	29	98.7%	377	29	58685	36908	36403	1679	99.3%	8739	1072	41521	17945	13188	246	99.7%	234	14
5017	2yr	S3	16131	880	762	30	99.1%	370	23	49978	30799	30368	1679	99.1%	9026	1395	79409	37883	27782	343	99.9%	239	16
5020	2w	F	64498	6	4	1	100.0%	375	1	117917	84246	83968	314	99.9%	8972	127							
5020	4w	F	45606	102	87	3	98.9%	374	3	58209	47658	47405	298	99.9%	8992	186							
5020	8w	F	12726	1617	1462	8	100.0%	376	8	51296	39302	39120	389	99.9%	8965	259							
5020	1yr	F	22561	3766	3381	35	99.9%	371	18	88062	58330	57580	1881	99.6%	8839	1064	41719	25572	21403	210	99.9%	241	17
5020	2yr	F	18336	2650	2315	30	99.7%	374	19	48062	30800	30442	1320	99.3%	9023	1110	18586	7984	5582	321	99.7%	208	42
5026	2w	F	70435	31	24	2	100.0%	375	2	36807	30376	30207	135	99.9%	9001	106							
5026	4w	F	47052	10	6	3	83.3%	376	3	59378	42440	42295	261	99.9%	8970	156							
5026	8w	F	12514	202	167	5	98.8%	374	5	74621	43291	43123	192	99.9%	8979	117							
5026	1yr	F	13404	1635	1429	25	99.8%	376	22	39478	26135	25726	1284	99.3%	8963	1099	50137	33564	25251	324	99.9%	243	34
5026	2yr	F	23101	1222	1091	26	99.8%	375	24	62225	44153	43559	1308	99.5%	8900	838	37535	21500	15797	832	99.8%	192	55

5031	2w	F	71679	11	8	4	75.0%	376	4	36417	30201	30066	123	99.9%	8998	98							
5031	4w	F	24146	837	725	9	99.7%	377	9	46997	35299	35176	234	99.8%	9005	155							
5031	8w	F	27747	782	701	10	99.6%	378	10	47400	35838	35528	470	99.8%	9036	386							
5031	1yr	F	15222	1253	1139	26	99.6%	374	21	36955	23368	22946	1413	99.0%	9005	1185	42489	19350	14447	678	99.9%	192	48
5031	1yr	L1	27417	1972	1728	44	99.7%	370	29	23213	15566	15104	1586	97.5%	9112	1586	167704	110562	82984	966	100.0%	230	17
5031	1yr	L2	18883	1472	1298	35	99.5%	372	28	27427	18742	18222	1457	98.0%	8657	1100	65558	36341	27852	366	99.9%	237	19
5031	1yr	L3	22499	1083	947	28	99.6%	375	24	50886	32442	31920	2048	98.9%	9108	1699	150573	97227	73001	851	100.0%	228	22
5031	1yr	S1	17521	1120	972	25	99.6%	373	21	69035	42868	41980	2469	99.2%	8835	1540	147310	86826	67983	630	100.0%	235	20
5031	1yr	S2	14363	515	447	17	99.1%	376	17	25695	16260	15856	1527	98.2%	9117	1527	57947	36025	28642	369	99.9%	235	19
5031	1yr	S3	25352	705	496	13	99.8%	377	13	25987	16211	15807	1547	98.1%	9101	1547	40263	25980	19115	406	99.8%	229	24
5031	2yr	F	27044	3419	2997	27	99.9%	376	17	50001	34827	34404	1090	99.5%	9078	914	59809	16677	11459	352	99.8%	225	29
5031	2yr	L1	18866	1826	1554	36	99.8%	370	28	72067	46093	45586	2169	99.3%	8914	1434	54348	23310	16943	272	99.8%	234	15
5031	2yr	L2	14579	1622	1445	39	99.7%	374	35	61196	40924	40286	2028	99.1%	8803	1266	58230	23847	17725	304	99.8%	237	15
5031	2yr	L3	11941	1277	1106	33	99.6%	375	29	57416	36052	35616	2043	99.1%	9235	1727	30043	12127	9239	184	99.8%	233	13
5031	2yr	S1	17428	693	615	24	99.7%	376	24	58121	37575	37109	1830	99.4%	8774	1239	46002	25660	20105	253	99.9%	239	15
5031	2yr	S2	17243	748	662	30	99.4%	377	30	66460	45524	44788	1919	99.2%	8859	1224	71123	31047	23454	382	99.8%	234	15
5031	2yr	S3	20299	803	702	27	99.1%	375	27	52994	35039	34627	1827	99.2%	9129	1563	35880	15360	11359	205	99.8%	234	14
5037	2w	F	67023	1	0		0.0%	0	0	92477	64881	64667	250	99.9%	8983	125							
5037	4w	F	16810	1704	1549	8	99.9%	373	5	51469	30448	30316	271	99.8%	8993	203							
5037	8w	F	24990	774	709	7	99.6%	377	7	57361	46172	45920	298	99.8%	8986	179							
5037	1yr	F	19627	1372	1216	22	99.8%	373	19	41807	27443	27015	1450	99.3%	9020	1252	59462	22582	17106	241	99.8%	238	36
5037	2yr	F	25533	2710	2368	21	99.9%	375	17	69258	50509	49877	1539	99.6%	8953	1030	55917	30629	23605	931	99.9%	189	55
5041	2w	F	68359	10	6	1	100.0%	375	1	25065	20300	20175	127	99.8%	8985	90							
5041	4w	F	21124	2393	2182	10	100.0%	374	7	32302	25482	25286	251	99.8%	8997	207							
5041	8w	F	24997	5285	4890	9	100.0%	374	4	70349	42749	42443	428	99.9%	8973	302							
5041	1yr	F	19064	1916	1665	26	99.9%	374	22	63924	42224	41540	1767	99.5%	8881	1201	45468	25916	19506	509	99.8%	225	29
5041	2yr	F	27020	2963	2656	28	99.8%	374	17	71800	52202	51591	1566	99.6%	8949	1057	63357	34669	25768	847	100.0%	201	75
5045	2w	F	64293	14	8	5	75.0%	376	5	33687	24749	24683	131	99.8%	8978	72							
5045	4w	F	18386	191	176	6	99.4%	376	6	38768	30880	30716	204	99.9%	9006	162							
5045	8w	F	62487	8442	7776	12	100.0%	375	5	78256	58706	58437	443	99.9%	8964	258							
5045	1yr	F	36692	5519	5068	40	99.9%	373	20	110388	74537	73508	1871	99.7%	8787	942	81171	57656	46231	434	99.9%	241	24
5045	1yr	L1	27034	1675	1443	37	99.7%	375	32	24355	14777	14348	1511	97.4%	9117	1511	80484	43126	32646	556	99.8%	234	27
5045	1yr	L2	26272	2097	1843	47	99.6%	371	34	31814	20283	19782	1597	98.2%	8816	1241	145855	73876	56200	800	99.9%	229	26
5045	1yr	L3	24967	1058	945	35	99.6%	373	31	35117	22636	22093	1507	98.5%	8895	1177	86752	38561	28724	524	99.8%	228	27
5045	1yr	S1	19950	556	475	23	99.6%	378	23	27401	17434	17033	1508	98.1%	9223	1508	78785	41161	31657	462	99.9%	233	25
5045	1yr	S2	22126	980	833	31	99.6%	375	28	29893	18642	18247	1466	98.7%	8686	1224	32892	14776	11538	263	99.7%	234	24
5045	1yr	S3	19592	867	766	27	99.6%	373	24	31104	20742	20177	1547	98.4%	8862	1224	68651	32702	25074	395	99.8%	236	27
5045	2yr	F	17138	1169	1005	20	99.7%	373	17	81048	57228	56507	1210	99.7%	8862	690	56663	27022	17360	334	99.9%	227	35
5045	2yr	L1	24749	3085	2745	52	99.9%	374	34	110192	72030	71200	2243	99.5%	8916	1236	85590	46355	35222	439	99.9%	238	14
5045	2yr	L2	24960	3737	3358	53	99.9%	372	34	122204	78062	76976	2305	99.6%	8804	1190	71233	29670	20378	355	99.9%	236	16
5045	2yr	L3	13422	1286	1110	26	99.8%	376	24	91177	59138	58357	2170	99.4%	8801	1216	51528	22318	16093	271	99.9%	236	16
5045	2yr	S1	16629	721	633	26	99.1%	376	26	121260	80577	79735	2027	99.6%	8803	989	72337	40445	29811	274	99.9%	243	14
5045	2yr	S2	28208	1903	1688	36	99.8%	374	27	91148	60050	59621	1792	99.6%	8829	989	96878	38864	29389	368	99.9%	240	18
5045	2yr	S3	19458	930	805	30	99.3%	372	24	85861	54895	54445	1821	99.6%	8752	1031	98177	35611	26651	373	99.9%	239	18
5046	2w	F	57291	5	1	1	0.0%	375	1	33647	22108	22003	172	99.7%	8978	104							
5046	4w	F	28313	786	721	7	100.0%	376	7	42886	29153	28991	274	99.7%	8999	201							

5046	1yr	F	16284	1245	1132	21	99.7%	374	18	100939	66154	65256	1978	99.5%	8859	1109	37552	21644	17025	281	99.8%	240	23
5046	2yr	F	47681	3957	3421	29	99.9%	372	15	62433	40583	40154	1501	99.5%	8879	1037	33047	19716	15193	744	99.9%	193	61
5049	2w	F	67782	2	1	1	0.0%	375	1	25913	17707	17620	102	99.8%	9019	102							
5049	4w	F	20315	763	694	4	100.0%	375	4	47350	37169	36971	273	99.9%	8966	176							
5049	1yr	F	15130	2762	2315	47	99.9%	371	33	49716	29830	29076	1745	99.4%	9143	1560	49963	32931	25559	389	99.9%	241	27
5049	2yr	F	51016	6414	5792	42	99.9%	370	14	76438	50663	50129	1723	99.6%	8959	1192	72212	33147	24720	892	99.9%	195	46
5053	2w	F	52388	48	28	3	92.9%	374	3	24309	17900	17835	130	99.7%	9035	130							
5053	4w	F	10664	345	301	6	99.3%	374	6	41589	31268	31097	386	99.8%	9019	333							
5053	8w	F	17300	931	842	7	99.8%	373	5	73452	56218	55899	455	99.9%	8945	248							
5053	1yr	F	33165	4515	4117	40	99.8%	371	17	81913	53224	52444	1866	99.5%	8945	1239	45940	27742	22526	273	99.9%	243	25
5053	1yr	L1	28920	3197	2836	46	99.8%	373	35	37587	24165	23553	1743	98.5%	8992	1398	85305	35980	28322	440	99.8%	236	26
5053	1yr	L2	32212	4168	3683	44	99.9%	373	31	24347	14966	14639	1517	97.7%	9034	1517	38044	18301	13775	306	99.8%	233	26
5053	1yr	L3	21044	2322	2016	42	99.8%	374	31	25542	15463	15157	1458	97.9%	9096	1458	37283	20033	14852	264	99.8%	239	25
5053	1yr	S1	24846	1309	1109	26	99.8%	375	24	15466	9507	9205	1241	96.9%	9091	1241	40554	19260	14338	286	99.8%	236	27
5053	1yr	S2	17703	822	704	27	99.3%	377	27	39899	25445	24797	1839	98.5%	8916	1464	67289	32212	24140	377	99.9%	238	27
5053	1yr	S3	16405	838	703	29	99.0%	380	29	35359	23749	23228	1787	98.3%	8945	1399	84060	45842	34977	469	99.9%	234	22
5053	2yr	F	14231	2430	2151	29	99.7%	372	19	59109	37983	37534	1239	99.5%	8859	820	16798	7946	5616	204	99.6%	228	27
5053	2yr	L1	17673	2800	2386	39	100.0%	371	24	53884	32939	32440	1850	99.2%	9095	1579	55563	20496	15024	340	99.8%	228	17
5053	2yr	L2	16144	2222	1879	48	99.6%	370	28	113902	74259	73251	2497	99.5%	8704	1263	79947	32063	23573	405	99.9%	233	18
5053	2yr	L3	12930	659	413	26	99.3%	378	26	177596	115126	114241	2869	99.7%	8735	1321	47070	17438	12283	310	99.8%	232	19
5053	2yr	S1	12933	696	598	23	99.3%	376	23	187083	128311	127434	2666	99.8%	8764	1231	80892	35845	24103	475	99.9%	231	20
5053	2yr	S2	14938	1092	959	30	99.8%	378	28	58231	37487	37006	1833	99.4%	8743	1197	83817	37411	26684	523	99.9%	226	16
5053	2yr	S3	14339	980	870	28	99.7%	373	25	54019	34693	34229	1817	99.2%	9136	1540	101954	43446	32177	531	99.9%	233	22
5058	2w	F	71673	24	19	1	100.0%	375	1	25159	18367	18269	75	99.9%	8984	50							
5058	4w	F	62362	80	64	3	98.4%	375	3	27546	20504	20392	235	99.8%	8989	198							
5058	1yr	F	35524	8440	7759	33	100.0%	372	15	91916	62211	61291	1990	99.5%	8827	1113	57997	33538	26944	216	99.9%	243	23
5058	2yr	F	16938	2057	1801	31	99.8%	371	22	41371	27847	27456	1165	99.3%	9026	978	30778	16586	11670	684	99.9%	192	62
5063	2w	F	23719	312	283	4	100.0%	374	4	43038	35337	35117	169	99.9%	9007	132							
5063	4w	F	81657	15	9	3	88.9%	375	3	56178	42105	41916	224	99.9%	8984	151							
5063	1yr	F	27901	4285	3904	24	99.9%	371	13	44457	29980	29488	1671	99.2%	9093	1423	66270	41536	33526	292	99.9%	238	15
5063	2yr	F	25328	3811	3354	26	99.9%	374	18	79341	56762	55982	1500	99.6%	8844	866	33499	18325	13944	550	99.9%	210	47

Type: feces (F), rumen liquids (L), rumen solids (S), numbers indicate day during 3 consecutive days of sampling
Raw: numer of contigs obtained off the MiSeq before any clean-up
Clean: numer of contigs after clean-up but before singleton removal
Abund: numer of contigs after clean-up and singleton removal
OTUs: number of OTUs among abund contigs
Coverage: Good's coverage calculated from abund contigs
Norm: number of contigs after normalization
Norm OTUs: number of OTUs among norm contigs

Table S5. P-values associated with statistical analyses of age groups, animal growth, health, and control variables.

							POST FDR-CORRECTION									
Test	Community	Type	Metric	AgeGroup	Animal	Scours	Random	2w-4w	4w-8w	8w-1yr	1yr-2yr	2w	4w	8w	1yr	2yr
ANOSIM	Archaea	Feces	Bray-Curtis	2.67E-04	1.000	1.000	0.795									
		Feces	Jaccard	2.67E-04	1.000	1.000	0.795									
		Liquids	Bray-Curtis	2.67E-04			1.000									
		Liquids	Jaccard	2.67E-04			1.000									
		Solids	Bray-Curtis	2.67E-04			1.000									
		Solids	Jaccard	2.67E-04			1.000									
	Bacteria	Feces	Bray-Curtis	2.67E-04	1.000	0.009	1.000									
		Feces	Jaccard	2.67E-04	1.000	0.008	1.000									
		Feces.2week	Bray-Curtis			0.972										
		Feces.2week	Jaccard			0.972										
		Liquids	Bray-Curtis	2.67E-04			1.000									
		Liquids	Jaccard	2.67E-04			1.000									
		Solids	Bray-Curtis	2.67E-04			1.000									
		Solids	Jaccard	2.67E-04			1.000									
	Fungi	Feces	Bray-Curtis	2.67E-04	1.000	NA	1.000									
		Feces	Jaccard	2.67E-04	1.000	NA	1.000									
		Liquids	Bray-Curtis	2.67E-04			1.000									
		Liquids	Jaccard	2.67E-04			1.000									
		Solids	Bray-Curtis	2.67E-04			0.517									
		Solids	Jaccard	2.67E-04			0.517									
ANOVA	Archaea	Feces	Shannon	1.44E-15			0.979									
		Feces	Chao	1.44E-15			0.979									
		Liquids	Shannon	1.44E-15			0.979									
		Liquids	Chao	4.16E-05			0.979									
		Solids	Shannon	7.98E-14			0.979									
		Solids	Chao	0.074			0.891									
	Bacteria	Feces	Shannon	1.44E-15			0.987									
		Feces	Chao	1.44E-15			0.979									
		Liquids	Shannon	1.89E-13			0.979									
		Liquids	Chao	5.92E-05			0.979									
		Solids	Shannon	4.59E-12			0.979									
		Solids	Chao	6.69E-05			0.979									
	Fungi	Feces	Shannon	0.003			0.673									
		Feces	Chao	5.92E-05			0.979									
		Liquids	Shannon	9.06E-05			0.979									
		Liquids	Chao	0.023			0.979									
		Solids	Shannon	0.040			0.979									
		Solids	Chao	0.438			0.891									
TukeyHSD	Archaea	Feces	Shannon					0.678	0.054	< 1E-08	0.605					
		Feces	Chao					0.498	0.990	< 1E-08	0.742					
		Liquids	Shannon							< 1E-08	0.096					
		Liquids	Chao							1.01E-05	0.926					
		Solids	Shannon							< 1E-08	0.004					
		Solids	Chao							0.180	0.351					
	Bacteria	Feces	Shannon					2.40E-04	2.06E-05	< 1E-08	0.328					
		Feces	Chao					0.582	0.957	< 1E-08	0.053					
		Liquids	Shannon							< 1E-08	0.966					
		Liquids	Chao							3.10E-05	0.926					
		Solids	Shannon							< 1E-08	0.001					
		Solids	Chao							2.41E-05	0.979					

	Fungi	Feces	Shannon				NA	0.001					
		Feces	Chao				NA	1.81E-05					
		Liquids	Shannon				5.89E-05	0.003					
		Liquids	Chao				0.996	0.010					
		Solids	Shannon				0.673	0.013					
		Solids	Chao				0.793	0.364					
PERMDISP	Archaea	Feces	Bray-Curtis	0.020	0.794	0.007	4.13E-05	0.170					
		Liquids	Bray-Curtis	3.33E-04			1.43E-08	1.74E-09					
		Solids	Bray-Curtis	0.013			0.019	0.063					
	Bacteria	Feces	Bray-Curtis	3.33E-04	0.017	0.104	9.08E-08	0.336					
		Liquids	Bray-Curtis	3.33E-04			0.027	8.18E-07					
		Solids	Bray-Curtis	0.003			0.016	4.21E-05					
	Fungi	Feces	Bray-Curtis	0.104			NA	0.104					
		Liquids	Bray-Curtis	9.00E-04			0.134	0.003					
		Solids	Bray-Curtis	0.029			0.209	0.098					
PERMANOVA, ADG weight	Archaea	Feces	Bray-Curtis						0.298	0.941	0.727	0.941	0.941
		Feces	Jaccard						0.226	0.938	0.727	0.967	0.934
		Liquids	Bray-Curtis								0.346	0.012	0.087
		Liquids	Jaccard								0.346	0.009	0.087
		Solids	Bray-Curtis								0.870	0.163	0.080
		Solids	Jaccard								0.870	0.080	0.080
	Bacteria	Feces	Bray-Curtis						0.941	0.080	0.980	0.941	0.938
		Feces	Jaccard						0.941	0.080	0.980	0.941	0.938
		Liquids	Bray-Curtis								0.789	0.087	0.012
		Liquids	Jaccard								0.789	0.080	0.020
		Solids	Bray-Curtis								0.789	0.072	0.080
		Solids	Jaccard								0.789	0.060	0.080
	Fungi	Feces	Bray-Curtis									0.107	0.870
		Feces	Jaccard									0.087	0.870
		Liquids	Bray-Curtis								0.870	0.053	0.380
		Liquids	Jaccard								0.870	0.026	0.309
		Solids	Bray-Curtis								0.870	0.012	0.726
		Solids	Jaccard								0.870	0.009	0.619
PERMANOVA, scours	Archaea	Feces	Bray-Curtis						0.822	0.513	0.513	NA	NA
		Feces	Jaccard						0.714	0.513	0.513	NA	NA
		Liquids	Bray-Curtis								0.714	NA	NA
		Liquids	Jaccard								0.714	NA	NA
		Solids	Bray-Curtis								0.513	NA	NA
		Solids	Jaccard								0.513	NA	NA
	Bacteria	Feces	Bray-Curtis						0.513	0.529	0.566	NA	NA
		Feces	Jaccard						0.513	0.566	0.566	NA	NA
		Liquids	Bray-Curtis								0.513	NA	NA
		Liquids	Jaccard								0.513	NA	NA
		Solids	Bray-Curtis								0.513	NA	NA
		Solids	Jaccard								0.513	NA	NA
	Fungi	Feces	Bray-Curtis						NS	NS	NS	NA	NA
		Feces	Jaccard						NS	NS	NS	NA	NA
		Liquids	Bray-Curtis								0.513	NA	NA
		Liquids	Jaccard								0.513	NA	NA
		Solids	Bray-Curtis								0.513	NA	NA
		Solids	Jaccard								0.513	NA	NA
		Feces	Bray-Curtis						0.255	0.255	0.361	NA	NA
		Feces	Jaccard						0.255	0.261	0.361	NA	NA

PERMANOVA, resp	Archaea	Liquids	Bray-Curtis				NV	NA	NA
		Liquids	Jaccard				NV	NA	NA
		Solids	Bray-Curtis				NV	NA	NA
		Solids	Jaccard				NV	NA	NA
	Bacteria	Feces	Bray-Curtis		0.255	0.255	0.788	NA	NA
		Feces	Jaccard		0.255	0.255	0.788	NA	NA
		Liquids	Bray-Curtis				NV	NA	NA
		Liquids	Jaccard				NV	NA	NA
		Solids	Bray-Curtis				NV	NA	NA
		Solids	Jaccard				NV	NA	NA
	Fungi	Feces	Bray-Curtis		NS	NS	NS	NA	NA
		Feces	Jaccard		NS	NS	NS	NA	NA
		Liquids	Bray-Curtis				NV	NA	NA
		Liquids	Jaccard				NV	NA	NA
		Solids	Bray-Curtis				NV	NA	NA
		Solids	Jaccard				NV	NA	NA
PERMANOVA, illness all	Archaea	Feces	Bray-Curtis		0.624	0.624	0.624	0.819	0.819
		Feces	Jaccard		0.624	0.624	0.624	0.819	0.819
		Liquids	Bray-Curtis				0.817	0.624	0.819
		Liquids	Jaccard				0.817	0.624	0.819
		Solids	Bray-Curtis				0.624	0.624	0.624
		Solids	Jaccard				0.624	0.624	0.624
	Bacteria	Feces	Bray-Curtis		0.817	0.624	0.801	0.624	0.649
		Feces	Jaccard		0.819	0.624	0.801	0.624	0.649
		Liquids	Bray-Curtis				0.624	0.819	0.817
		Liquids	Jaccard				0.624	0.819	0.817
		Solids	Bray-Curtis				0.624	0.705	0.695
		Solids	Jaccard				0.624	0.705	0.695
	Fungi	Feces	Bray-Curtis					0.624	0.705
		Feces	Jaccard					0.624	0.716
		Liquids	Bray-Curtis				0.624	0.917	0.624
		Liquids	Jaccard				0.624	0.917	0.624
		Solids	Bray-Curtis				0.624	0.817	0.624
		Solids	Jaccard				0.624	0.817	0.624

NS: not sequenced; NV: no variation in variable of interest; NA: not applicable
Significant P-values (P < 0.05) are in red

Test	Community	Type	Metric	AgeGroup	Animal	Scours	Random	RAW, NO FDR-CORRECTION								
								2w-4w	4w-8w	8w-1yr	1yr-2yr	2w	4w	8w	1yr	2yr
ANOSIM	Archaea	Feces	Bray-Curtis	1.00E-04	0.959	0.871	0.396									
		Feces	Jaccard	1.00E-04	0.956	0.877	0.398									
		Liquids	Bray-Curtis	1.00E-04			0.668									
		Liquids	Jaccard	1.00E-04			0.670									
		Solids	Bray-Curtis	1.00E-04			0.890									
		Solids	Jaccard	1.00E-04			0.895									
	Bacteria	Feces	Bray-Curtis	1.00E-04	1.000	0.004	0.914									
		Feces	Jaccard	1.00E-04	1.000	0.003	0.904									
		Feces.2week	Bray-Curtis			0.526										
		Feces.2week	Jaccard			0.525										
		Liquids	Bray-Curtis	1.00E-04			0.791									
		Liquids	Jaccard	1.00E-04			0.785									
		Solids	Bray-Curtis	1.00E-04			0.704									
		Solids	Jaccard	1.00E-04			0.701									
	Fungi	Feces	Bray-Curtis	1.00E-04	0.999	NA	0.900									
		Feces	Jaccard	1.00E-04	0.998	NA	0.901									
		Liquids	Bray-Curtis	1.00E-04			0.635									
		Liquids	Jaccard	1.00E-04			0.629									
		Solids	Bray-Curtis	1.00E-04			0.233									
		Solids	Jaccard	1.00E-04			0.237									
ANOVA	Archaea	Feces	Shannon	< 2E-16			0.944									
		Feces	Chao	< 2E-16			0.662									
		Liquids	Shannon	< 2E-16			0.952									
		Liquids	Chao	1.04E-05			0.714									
		Solids	Shannon	1.33E-14			0.858									
		Solids	Chao	0.035			0.520									
	Bacteria	Feces	Shannon	< 2E-16			0.987									
		Feces	Chao	< 2E-16			0.850									
		Liquids	Shannon	3.67E-14			0.763									
		Liquids	Chao	1.78E-05			0.740									
		Solids	Shannon	1.02E-12			0.729									
		Solids	Chao	2.23E-05			0.946									
	Fungi	Feces	Shannon	0.001			0.355									
		Feces	Chao	1.81E-05			0.778									
		Liquids	Shannon	3.27E-05			0.952									
		Liquids	Chao	0.010			0.714									
		Solids	Shannon	0.018			0.858									
		Solids	Chao	0.219			0.520									
TukeyHSD	Archaea	Feces	Shannon					0.678	0.054	< 1E-08	0.605					
		Feces	Chao					0.498	0.990	< 1E-08	0.742					
		Liquids	Shannon							< 1E-08	0.096					
		Liquids	Chao							1.01E-05	0.926					
		Solids	Shannon							< 1E-08	0.004					
		Solids	Chao							0.180	0.351					
	Bacteria	Feces	Shannon					2.40E-04	2.06E-05	< 1E-08	0.328					
		Feces	Chao					0.582	0.957	< 1E-08	0.053					
		Liquids	Shannon							< 1E-08	0.966					
		Liquids	Chao							3.10E-05	0.926					
		Solids	Shannon							< 1E-08	0.001					
		Solids	Chao							2.41E-05	0.979					

	Fungi	Feces	Shannon				NA	0.001					
		Feces	Chao				NA	1.81E-05					
		Liquids	Shannon				5.89E-05	0.003					
		Liquids	Chao				0.996	0.010					
		Solids	Shannon				0.673	0.013					
		Solids	Chao				0.793	0.364					
PERMDISP	Archaea	Feces	Bray-Curtis	0.012	0.794	0.003	6.88E-06	0.153					
		Liquids	Bray-Curtis	1.00E-04			9.55E-10	5.80E-11					
		Solids	Bray-Curtis	0.006			0.011	0.044					
	Bacteria	Feces	Bray-Curtis	1.00E-04	0.009	0.080	9.08E-09	0.325					
		Liquids	Bray-Curtis	1.00E-04			0.017	1.09E-07					
		Solids	Bray-Curtis	0.001			0.008	8.42E-06					
	Fungi	Feces	Bray-Curtis	0.087			NA	0.083					
		Liquids	Bray-Curtis	3.00E-04			0.116	0.001					
		Solids	Bray-Curtis	0.019			0.195	0.072					
PERMANOVA, ADG weight	Archaea	Feces	Bray-Curtis						0.129	0.824	0.412	0.894	0.802
		Feces	Jaccard						0.094	0.779	0.404	0.935	0.732
		Liquids	Bray-Curtis								0.167	0.001	0.030
		Liquids	Jaccard								0.167	0.000	0.032
		Solids	Bray-Curtis								0.667	0.065	0.024
		Solids	Jaccard								0.667	0.023	0.018
	Bacteria	Feces	Bray-Curtis						0.849	0.016	0.980	0.882	0.782
		Feces	Jaccard						0.865	0.017	0.980	0.870	0.774
		Liquids	Bray-Curtis								0.500	0.030	0.001
		Liquids	Jaccard								0.500	0.022	0.002
		Solids	Bray-Curtis								0.500	0.012	0.022
		Solids	Jaccard								0.500	0.009	0.019
	Fungi	Feces	Bray-Curtis						NS	NS	NS	0.041	0.639
		Feces	Jaccard						NS	NS	NS	0.031	0.585
		Liquids	Bray-Curtis								0.667	0.007	0.190
		Liquids	Jaccard								0.667	0.003	0.139
		Solids	Bray-Curtis								0.667	0.001	0.387
		Solids	Jaccard								0.667	0.000	0.320
PERMANOVA, scours	Archaea	Feces	Bray-Curtis						0.822	0.228	0.062	NA	NA
		Feces	Jaccard						0.684	0.342	0.059	NA	NA
		Liquids	Bray-Curtis								0.667	NA	NA
		Liquids	Jaccard								0.667	NA	NA
		Solids	Bray-Curtis								0.333	NA	NA
		Solids	Jaccard								0.333	NA	NA
	Bacteria	Feces	Bray-Curtis						0.310	0.375	0.463	NA	NA
		Feces	Jaccard						0.327	0.471	0.472	NA	NA
		Liquids	Bray-Curtis								0.333	NA	NA
		Liquids	Jaccard								0.333	NA	NA
		Solids	Bray-Curtis								0.333	NA	NA
		Solids	Jaccard								0.333	NA	NA
	Fungi	Feces	Bray-Curtis						NS	NS	NS	NA	NA
		Feces	Jaccard						NS	NS	NS	NA	NA
		Liquids	Bray-Curtis								0.333	NA	NA
		Liquids	Jaccard								0.333	NA	NA
		Solids	Bray-Curtis								0.333	NA	NA
		Solids	Jaccard								0.333	NA	NA
		Feces	Bray-Curtis						0.102	0.132	0.301	NA	NA
		Feces	Jaccard						0.109	0.174	0.288	NA	NA

PERMANOVA, resp	Archaea	Liquids	Bray-Curtis				NV	NA	NA
		Liquids	Jaccard				NV	NA	NA
		Solids	Bray-Curtis				NV	NA	NA
		Solids	Jaccard				NV	NA	NA
	Bacteria	Feces	Bray-Curtis		0.041	0.123	0.788	NA	NA
		Feces	Jaccard		0.047	0.149	0.773	NA	NA
		Liquids	Bray-Curtis				NV	NA	NA
		Liquids	Jaccard				NV	NA	NA
		Solids	Bray-Curtis				NV	NA	NA
		Solids	Jaccard				NV	NA	NA
	Fungi	Feces	Bray-Curtis		NS	NS	NS	NA	NA
		Feces	Jaccard		NS	NS	NS	NA	NA
		Liquids	Bray-Curtis				NV	NA	NA
		Liquids	Jaccard				NV	NA	NA
		Solids	Bray-Curtis				NV	NA	NA
		Solids	Jaccard				NV	NA	NA
PERMANOVA, illness all	Archaea	Feces	Bray-Curtis		0.296	0.055	0.065	0.788	0.738
		Feces	Jaccard		0.267	0.113	0.061	0.785	0.754
		Liquids	Bray-Curtis				0.667	0.167	0.792
		Liquids	Jaccard				0.667	0.167	0.792
		Solids	Bray-Curtis				0.333	0.292	0.125
		Solids	Jaccard				0.333	0.292	0.125
	Bacteria	Feces	Bray-Curtis		0.655	0.061	0.561	0.266	0.368
		Feces	Jaccard		0.683	0.099	0.553	0.276	0.360
		Liquids	Bray-Curtis				0.333	0.792	0.625
		Liquids	Jaccard				0.333	0.792	0.625
		Solids	Bray-Curtis				0.333	0.458	0.417
		Solids	Jaccard				0.333	0.458	0.417
	Fungi	Feces	Bray-Curtis					0.310	0.437
		Feces	Jaccard					0.330	0.477
		Liquids	Bray-Curtis				0.333	0.917	0.208
		Liquids	Jaccard				0.333	0.917	0.208
		Solids	Bray-Curtis				0.333	0.667	0.042
		Solids	Jaccard				0.333	0.667	0.042

Table S6. OTUs which contribute to differences between age groups (SIMPER) and/or have strong correlations (Kendall) to animal growth or health.

			Contribution to SIMPER (%)										Kendall correlation		Classification	
Amplicon	Type	OTU	2w-4w	2w-8w	2w-1yr	2w-2yr	4w-8w	4w-1yr	4w-2yr	8w-1yr	8w-2yr	1yr-2yr	1yr-ADG	2yr-ADG	Phyla	Lowest
		OTU01	39.7%	47.6%	34.2%	34.6%	48.0%	33.2%	35.2%	47.5%	47.5%				Euryarchaeota	*Methanobrevibacter*
		OTU02		11.8%	7.6%	12.4%		6.1%	12.6%		11.7%	12.0%			Euryarchaeota	*Methanobrevibacter*
		OTU03	16.6%	19.5%	14.5%	9.3%		11.5%		10.3%		11.6%			Euryarchaeota	*Methanobrevibacter*
	Feces	OTU04			18.8%			18.3%		17.7%		23.8%			Euryarchaeota	Methanocorpusculaceae
		OTU05	11.4%			12.1%	16.0%		12.7%		11.8%	16.9%			Euryarchaeota	*Methanobrevibacter*
		OTU09				8.8%			9.0%			11.3%			Euryarchaeota	*Methanobrevibacter*
		OTU17	11.6%				15.8%	6.5%	6.9%						Euryarchaeota	*Methanosphaera*
	Feces	cumulative	79.4%	78.9%	75.1%	77.2%	79.8%	75.5%	76.3%	75.6%	71.0%	75.5%				
		OTU01								49.6%	49.5%				Euryarchaeota	*Methanobrevibacter*
		OTU02									10.3%	13.4%			Euryarchaeota	*Methanobrevibacter*
		OTU03								7.0%		6.3%	-0.78		Euryarchaeota	*Methanobrevibacter*
		OTU05										5.7%			Euryarchaeota	*Methanobrevibacter*
	Liquids	OTU06								8.9%		13.1%			Euryarchaeota	*Methanobrevibacter*
		OTU07								6.3%	7.4%	8.7%			Euryarchaeota	*Methanobrevibacter*
Archaea		OTU08										10.0%			Euryarchaeota	*Methanobrevibacter*
		OTU09									9.7%	16.9%			Euryarchaeota	*Methanobrevibacter*
		OTU25											0.79		Euryarchaeota	*vadinCA11*
		OTU36											0.80		Euryarchaeota	*Methanobrevibacter*
	Liquids	cumulative								71.7%	76.9%	74.0%				
		OTU01								47.8%	47.9%				Euryarchaeota	*Methanobrevibacter*
		OTU02								10.7%		11.7%			Euryarchaeota	*Methanobrevibacter*
		OTU03								17.5%	12.8%	12.3%			Euryarchaeota	*Methanobrevibacter*
		OTU05									10.6%	22.4%			Euryarchaeota	*Methanobrevibacter*
	Solids	OTU08										6.0%			Euryarchaeota	*Methanobrevibacter*
		OTU11										7.8%			Euryarchaeota	*Methanobrevibacter*
		OTU13										3.7%			Euryarchaeota	*Methanobrevibacter*
		OTU16										5.8%			Euryarchaeota	*vadinCA11*
		OTU25										3.1%			Euryarchaeota	*vadinCA11*
	Solids	cumulative								76.0%	71.3%	72.9%				
		OTU001	10.8%	5.4%	4.3%	4.3%	10.9%	10.0%	10.0%	2.6%	2.7%				Firmicutes	*Faecalibacterium*
		OTU003	9.2%	6.2%	5.6%	5.6%	6.5%	5.6%	5.6%						Firmicutes	*Faecalibacterium*
		OTU006	7.2%	6.4%	6.0%	6.0%	2.9%	2.5%	2.6%						Actinobacteria	*Collinsella*
		OTU008	4.3%	3.9%	3.6%	3.6%									Actinobacteria	*Bifidobacterium*
		OTU009	1.8%	1.7%			2.0%	1.5%	1.5%	1.6%	1.7%				Bacteroidetes	*Bacteroides*
		OTU010	3.1%	3.9%	2.6%	2.6%	1.7%			1.4%	1.4%				Firmicutes	*Clostridium*
		OTU012			1.7%	2.1%		1.7%	2.1%	1.5%	1.9%	1.1%			Firmicutes	Ruminococcaceae
		OTU013	1.0%	4.0%			4.5%			3.7%	3.8%				Bacteroidetes	S24-7
		OTU015	3.7%	3.6%	3.4%	3.4%	1.2%								Firmicutes	Ruminococcaceae
		OTU016	1.8%	2.2%	1.5%	1.5%	1.3%			1.1%	1.1%				Proteobacteria	Enterobacteriaceae
		OTU017	2.9%	2.8%			4.1%	2.0%	2.0%	2.3%	2.3%				Bacteroidetes	*Bacteroides*
		OTU018	4.0%	1.1%			4.4%	3.3%	3.3%	1.0%	1.1%				Bacteroidetes	*Bacteroides*
		OTU020	2.2%	1.1%			1.6%	1.3%	1.3%						Bacteroidetes	*Bacteroides*
		OTU024			1.5%	1.1%		1.5%	1.1%	1.6%	1.1%	1.1%			Bacteroidetes	*5-7N15*
		OTU025	3.7%	3.4%	3.2%	3.2%									Firmicutes	*Dorea*
		OTU026	3.1%	2.8%	2.6%	2.6%									Firmicutes	*Lactobacillus*

		OTU028				2.3%			2.3%		2.4%	3.8%		Spirochaetes	*Treponema*
	Feces	OTU030	1.4%				1.3%	1.2%	1.2%					Bacteroidetes	*Bacteroides*
		OTU032			1.4%			1.4%		1.4%		1.7%		Bacteroidetes	Bacteroidaceae
		OTU034	1.3%	1.0%			1.1%							Firmicutes	Ruminococcaceae
		OTU035	1.6%	1.4%	1.0%	1.0%								Firmicutes	*Blautia*
		OTU038				1.0%			1.0%		1.1%			Bacteroidetes	*CF231*
		OTU043				1.3%			1.3%		1.4%	1.9%		Bacteroidetes	S24-7
		OTU046				1.3%			1.6%		1.3%	2.2%		Actinobacteria	*Bifidobacterium*
		OTU061										1.5%		Bacteroidetes	*CF231*
		OTU064	1.2%	1.1%										Bacteroidetes	*Prevotella*
		OTU067										1.0%		Bacteroidetes	Bacteroidales
		OTU075	1.5%				1.5%	1.3%	1.3%					Firmicutes	*Blautia*
		OTU091										1.5%		Bacteroidetes	*CF231*
		OTU097										1.1%		Proteobacteria	*Succinivibrio*
		OTU101										1.1%		Bacteroidetes	*CF231*
		OTU106	1.1%											Firmicutes	*Blautia*
		OTU122					1.1%							Spirochaetes	*Treponema*
		OTU152	2.1%	1.9%	1.8%	1.8%								Firmicutes	*Peptostreptococcus*
		OTU157					1.0%							Bacteroidetes	Prevotellaceae
		OTU170		1.3%			1.5%			1.3%	1.3%			Actinobacteria	Coriobacteriaceae
	Feces	cumulative	69.2%	55.1%	40.2%	44.8%	48.6%	33.3%	38.4%	19.6%	24.4%	18.1%			
		OTU002								2.7%	2.7%	2.1%		Proteobacteria	Succinivibrionaceae
		OTU004								3.1%	2.1%	2.9%		Bacteroidetes	*Prevotella*
		OTU005								3.4%	3.4%	2.6%		Firmicutes	*Succiniclasticum*
		OTU007								1.1%	3.2%	4.3%		Proteobacteria	Succinivibrionaceae
		OTU014								1.7%	2.0%	1.0%		Bacteroidetes	*Prevotella*
Bacteria		OTU019								2.7%		3.7%		Bacteroidetes	*Prevotella*
		OTU021									1.5%	2.2%		Bacteroidetes	*Prevotella*
		OTU022									1.4%	1.9%		Bacteroidetes	*Prevotella*
		OTU023								7.5%	7.6%			Bacteroidetes	*Prevotella*
		OTU031								2.0%		3.0%		Bacteroidetes	*Prevotella*
	Liquids	OTU039								1.1%	1.1%			Bacteroidetes	*Prevotella*
		OTU057								5.5%	5.6%			Bacteroidetes	*Prevotella*
		OTU068								1.2%	1.2%			Bacteroidetes	*Prevotella*
		OTU070										1.0%		Bacteroidetes	*Prevotella*
		OTU079								1.4%	1.4%			Firmicutes	*Acidaminococcus*
		OTU080								3.6%	3.7%			Firmicutes	*Succiniclasticum*
		OTU089								3.3%	3.4%			Firmicutes	*Ruminococcus*
		OTU123								2.2%	2.2%			Bacteroidetes	*Prevotella*
		OTU194											-0.74	Bacteroidetes	*Prevotella*
		OTU217								1.1%	1.2%			Bacteroidetes	*Prevotella*
		OTU235								1.3%	1.4%			Bacteroidetes	p-2534-18B5
		OTU354								1.0%	1.0%			Firmicutes	*Bulleidia*
	Liquids	cumulative								46.1%	46.0%	24.7%			
		OTU002								2.2%	4.2%	8.6%		Proteobacteria	Succinivibrionaceae
		OTU004								1.2%	1.6%			Bacteroidetes	*Prevotella*
		OTU005								2.3%	3.0%	1.5%		Firmicutes	*Succiniclasticum*
		OTU007									3.2%	4.4%		Proteobacteria	Succinivibrionaceae
		OTU014									1.5%			Bacteroidetes	*Prevotella*

	OTU019	2.1%		2.5%	Bacteroidetes	*Prevotella*
	OTU021		1.0%	1.5%	Bacteroidetes	*Prevotella*
	OTU022			1.4%	Bacteroidetes	*Prevotella*
	OTU023	9.0%	9.3%		Bacteroidetes	*Prevotella*
	OTU027	1.6%		1.9%	Firmicutes	*Succiniclasticum*
	OTU029			1.2%	Bacteroidetes	*Prevotella*
	OTU039	1.0%	1.1%		Bacteroidetes	*Prevotella*
Solids	OTU042	1.6%		2.3%	Bacteroidetes	Bacteroidales
	OTU057	4.0%	4.1%		Bacteroidetes	*Prevotella*
	OTU062	1.3%	1.3%		Bacteroidetes	*Prevotella*
	OTU068	2.5%	2.6%		Bacteroidetes	*Prevotella*
	OTU079	1.7%	1.8%		Firmicutes	*Acidaminococcus*
	OTU080	3.0%	3.1%		Firmicutes	*Succiniclasticum*
	OTU102	1.2%	1.2%		Firmicutes	*Megasphaera*
	OTU104	1.6%	1.6%		Firmicutes	Veillonellaceae
	OTU114			1.6%	Bacteroidetes	Bacteroidales
	OTU123	2.6%	2.7%		Bacteroidetes	*Prevotella*
	OTU221	1.1%	1.1%		Bacteroidetes	*Prevotella*
	OTU275	1.0%	1.1%		Firmicutes	*Roseburia*
	OTU277	1.1%	1.1%		Firmicutes	*Acidaminococcus*
Solids	cumulative	41.9%	46.9%	27.0%		
	OTU001			11.5%	Chytridiomycota	*Caecomyces*
	OTU002			7.5%	Chytridiomycota	*Caecomyces*
	OTU003			1.0%	Chytridiomycota	Neocallimastigaceae
	OTU004			3.2%	Chytridiomycota	*Caecomyces*
	OTU005			1.1%	Chytridiomycota	*Piromyces*
	OTU006			3.3%	Chytridiomycota	Neocallimastigaceae
	OTU007			6.2%	Chytridiomycota	*Caecomyces*
	OTU008			3.7%	Chytridiomycota	*Orpinomyces*
	OTU009			1.6%	Chytridiomycota	*Cyllamyces*
	OTU010			1.7%	Chytridiomycota	Neocallimastigaceae
Feces	OTU013			2.3%	Chytridiomycota	Neocallimastigaceae
	OTU017			8.6%	unclassified	
	OTU018			1.9%	Chytridiomycota	*Orpinomyces*
	OTU019			1.1%	Chytridiomycota	Neocallimastigaceae
	OTU022			1.6%	Chytridiomycota	*Orpinomyces*
	OTU023			1.0%	Chytridiomycota	*Orpinomyces*
	OTU027			1.6%	Chytridiomycota	*Cyllamyces*
	OTU039			1.9%	unclassified	
	OTU041			1.5%	unclassified	
	OTU044			1.1%	unclassified	
Feces	cumulative			63.6%		
	OTU001	4.3%	8.8%	8.4%	Chytridiomycota	*Caecomyces*
	OTU002	3.3%	7.1%	6.1%	Chytridiomycota	*Caecomyces*
	OTU003	3.8%	9.3%	9.4%	Chytridiomycota	Neocallimastigaceae
	OTU004		6.3%	8.5%	Chytridiomycota	*Caecomyces*
	OTU005	3.8%		5.5%	Chytridiomycota	*Piromyces*
	OTU006	2.7%	5.0%	4.3%	Chytridiomycota	Neocallimastigaceae
	OTU007	4.6%		3.8%	Chytridiomycota	*Caecomyces*

Fungi	Liquids	OTU008	4.5%		6.4%		Chytridiomycota	*Orpinomyces*
		OTU011			4.8%		Chytridiomycota	Neocallimastigaceae
		OTU014	2.6%		3.7%		Chytridiomycota	Neocallimastigaceae
		OTU015	3.4%		4.8%	0.88	Chytridiomycota	*Piromyces*
		OTU019			4.9%		Chytridiomycota	Neocallimastigomycetes
		OTU036				0.81	Chytridiomycota	Neocallimastigomycetes
		OTU037	13.6%	13.3%			unclassified	
		OTU046	10.5%	10.3%			unclassified	
		OTU058	4.0%	3.9%			Chytridiomycota	*Piromyces*
		OTU066	4.5%	4.4%			Chytridiomycota	*Caecomyces*
		OTU076	4.7%	4.6%			unclassified	
	Liquids	cumulative	70.2%	73.0%	70.6%			
	Solids	OTU001	5.6%	11.0%	9.7%		Chytridiomycota	*Caecomyces*
		OTU002	4.1%	7.9%	7.6%	0.81	Chytridiomycota	*Caecomyces*
		OTU003	5.0%	5.2%	6.7%		Chytridiomycota	Neocallimastigaceae
		OTU004		8.0%	10.2%		Chytridiomycota	*Caecomyces*
		OTU005	4.3%		6.1%		Chytridiomycota	*Piromyces*
		OTU006	2.2%	5.1%	5.3%		Chytridiomycota	Neocallimastigaceae
		OTU007	4.3%		6.1%		Chytridiomycota	*Caecomyces*
		OTU008	5.0%		7.2%		Chytridiomycota	*Orpinomyces*
		OTU010	2.1%				Chytridiomycota	Neocallimastigaceae
		OTU011		4.1%	5.2%		Chytridiomycota	Neocallimastigaceae
		OTU012	6.8%	7.2%			Chytridiomycota	*Caecomyces*
		OTU013	1.7%				Chytridiomycota	Neocallimastigaceae
		OTU015	2.3%		3.3%	0.83	Chytridiomycota	*Piromyces*
		OTU016				0.74	Chytridiomycota	*Piromyces*
		OTU019		3.4%	4.8%		Chytridiomycota	Neocallimastigaceae
		OTU036				0.89	Chytridiomycota	Neocallimastigaceae
		OTU037	5.5%	5.6%			unclassified	
		OTU040					Chytridiomycota	Neocallimastigaceae
		OTU046	7.7%	7.8%			unclassified	
		OTU066	2.3%				Chytridiomycota	*Caecomyces*
		OTU076	3.4%	3.4%			unclassified	
		OTU103	3.7%	3.8%			Chytridiomycota	*Caecomyces*
		OTU202	2.4%				unclassified	
		OTU360	2.2%				Chytridiomycota	*Caecomyces*
	Solids	cumulative	70.6%	72.4%	72.2%			

Only OTUs at 0.5% or greater in at least one sample are shown. Only SIMPER contributions > 1% and strong correlations (Kendall < -0.70 or > 0.70) are show

Text S1. Sequence clean-up and analysis in mothur v1.36.1 and analysis in R v3.2.3 (R markdown format)

<u>mothur v1.36.1</u>

Archaea:
A = 13862
B = 23444
C = silva.nr_v119.align
D = silva.nr_v119.tax
E = unknown;-Bacteria;-Eukaryota;
F = 375
G = gg_2013.fasta
H = gg_2013.gg.tax
I = Example.final.gg.wang.taxonomy

Bacteria:
A = 28439
B = 42539
C = silva.nr_v119.align
D = silva.nr_v119.tax
E = unknown;-Archaea;-Eukaryota;-Bacteria;Cyanobacteria;-Bacteria;Proteobacteria;Alphaproteobacteria;Rickettsiales;mitochondria;
F = 9000
G = gg_2013.fasta
H = gg_2013.gg.tax
I = Example.final.gg.wang.taxonomy

Fungi:
skip align.seqs, screen.seqs, filter.seqs, and unique.seqs
C,G = UNITEv6_sh_dynamic_s.fasta
D,H = UNITEv6_sh_dynamic_s.tax
E = unknown;-Archaea;-Bacteria;
F = 250
I = Example.final.UNITEv6_sh_dynamic_s.wang.taxonomy

<u>mothur clean-up</u>

```
make.file(inputdir=/Example)
make.contigs(file=Example.file)
screen.seqs(fasta=Example.trim.contigs.fasta, group=Example.contigs.groups, maxambig=0,
        maxhomop=8, minlength=200, maxlength=600)
unique.seqs(fasta=Example.trim.contigs.good.fasta)
count.seqs(name=Example.trim.contigs.good.names, group=Example.contigs.good.groups)
summary.seqs(fasta=Example.trim.contigs.good.unique.fasta,
        count=Example.trim.contigs.good.count_table)
align.seqs(fasta=Example.trim.contigs.good.unique.fasta, reference=silva.nr_v119.align, flip=T)
screen.seqs(fasta=Example.trim.contigs.good.unique.align,
        count=Example.trim.contigs.good.count_table,
        summary=Example.trim.contigs.good.unique.summary, start=A, end=B)
filter.seqs(fasta=Example.trim.contigs.good.unique.good.align, vertical=T, trump=.)
unique.seqs(fasta=Example.trim.contigs.good.unique.good.filter.fasta,
        count=Example.trim.contigs.good.good.count_table)
pre.cluster(fasta=Example.trim.contigs.good.unique.good.filter.unique.fasta,
        count=Example.trim.contigs.good.unique.good.filter.count_table, diffs=2)
```

```
chimera.uchime(fasta=Example.trim.contigs.good.unique.good.filter.unique.precluster.fasta,
        count=Example.trim.contigs.good.unique.good.filter.unique.precluster.count_table)
remove.seqs(fasta=Example.trim.contigs.good.unique.good.filter.unique.precluster.fasta,
        count=Example.trim.contigs.good.unique.good.filter.unique.precluster.count_table,
        accnos=Example.trim.contigs.good.unique.good.filter.unique.precluster.denovo.uchime.accnos)
classify.seqs(fasta=Example.trim.contigs.good.unique.good.filter.unique.precluster.pick.fasta,
        count=Example.trim.contigs.good.unique.good.filter.unique.precluster.pick.count_table,
        reference=C, taxonomy=D, cutoff=60)
remove.lineage(fasta=Example.trim.contigs.good.unique.good.filter.unique.precluster.pick.fasta,
        count=Example.trim.contigs.good.unique.good.filter.unique.precluster.pick.count_table,
        taxonomy=Example.trim.contigs.good.unique.good.filter.unique.precluster.pick.nr_v119.wang.ta
        xonomy, taxon=E)
count.groups(count=Example.trim.contigs.good.unique.good.filter.unique.precluster.pick.pick.count_tabl
        e)
split.abund(fasta=Example.trim.contigs.good.unique.good.filter.unique.precluster.pick.pick.fasta,
        count=Example.trim.contigs.good.unique.good.filter.unique.precluster.pick.pick.count_table,
        cutoff=1)
count.groups(count=Example.trim.contigs.good.unique.good.filter.unique.precluster.pick.pick.abund.cou
        nt_table)
system(cp Example.trim.contigs.good.unique.good.filter.unique.precluster.pick.pick.abund.fasta
        Example.final.fasta)
system(cp Example.trim.contigs.good.unique.good.filter.unique.precluster.pick.pick.abund.count_table
        Example.final.count_table)
```

<u>mothur analysis</u>

```
dist.seqs(fasta=Example.final.fasta)
cluster.split(column=Example.final.dist, count=Example.final.count_table, method=furthest, cutoff=0.05)
make.shared(list=Example.final.fn.unique_list.list, count=Example.final.count_table, label=0.03)
normalize.shared(shared=Example.final.fn.unique_list.shared, method=totalgroup, norm= F)

summary.single(shared=Example.final.fn.unique_list.shared, label=0.03, calc=nseqs-sobs-coverage)
summary.single(shared=Example.final.fn.unique_list.0.03.norm.shared, label=0.03, calc=chao-shannon)

classify.seqs(fasta=Example.final.fasta, count=Example.final.count_table, template= G, taxonomy= H,
        cutoff=80)
classify.otu(list=Example.final.fn.unique_list.list, taxonomy=I, count=Example.final.count_table,
        label=0.03, cutoff=80, basis=otu, probs=F)
```

<u>R analysis</u>

````
```{r Load data}
#Normalized OTU table
#rows = samples IDs, columns = OTUs, data=normalized counts
OTU = read.table("Normalized.OTUs.txt", header=TRUE, row.names=1, sep="/t")

#Variables and metrics of interest
#rows = samples IDs, columns = variables/metrics i.e. AgeGroup, Shannon's diversity, etc., data=varied
var = read.table("Variables.txt", header=TRUE, row.names=1, sep="/t")
```

```{r Community diversity and richness: ANOVA, TukeyHSD}
#EXAMPLE: test Shannon’s diversity metric between age groups
````
````

````
#ANOVA test for differences
anova.test = aov(shannon~AgeGroup, data=var)
summary(anova.test)

#Tukey’s honest significant difference (HSD) from multiple pairwise comparisons between age groups
TukeyHSD(anova.test)

#Boxplot of Shannon's by age group
plot(var$shannon~MetFA$AgeGroup, boxwex=0.75)
```


```{r Non-metric multidimensional scaling plots}
#Load packages
library(vegan)
library(plotrix)
library(TeachingDemos)

#Calculate Bray-Curtis distance matrix for OTU table
nmds=metaMDS(OTU, distance="bray", k=2, trymax=1000)

#Create color vector for continuous rainbow coloring by exact age in days
#Use the natural log of exact age (Daysln) to scale the colors properly
colvec=colorRampPalette(c('darkred', 'red', 'orangered', 'orange', 'yellow', 'lightgreen', 'green', 'darkgreen',
'cyan ', 'light blue', 'blue', 'darkblue', 'purple', 'pink'))
var$col=colvec(200)[as.numeric(cut(var$Daysln, breaks=200))]

#EXAMPLE: basic plot
#Plot the nmds with rainbow colors
plot(nmds, type="n")
points(nmds, pch=20, cex=2.5, display="sites", col=var$col)

#EXAMPLE: split x-axis plot
#Pull out x and y values for the nmds
nmds.xy=scores(nmds, display="sites")

#Specify which values are above or below the desired x-axis gap and scale them to reduce the gap size
#In this example, the first 3 samples are very different from the rest
xgap=ifelse(nmds.xy[,1]>nmds.xy[4,1]-1, (nmds.xy[,1]+(nmds.xy[3,1]-nmds.xy[4,1]+1.5)), nmds.xy[,1])

#Plot the nmds with new scaled x-axis values
plot(xgap, nmds.xy[,2], pch=20, cex=2.5, col=var$col, xaxt="n")

#Label with x-axis with the scaled values
xat = pretty(xgap)
xat = xat[xat!=nmds.xy[3,1]+0.6]
xlab = ifelse(xat>nmds.xy[3,1]+0.6, xat-(nmds.xy[3,1]-nmds.xy[4,1]+1.5), xat)
axis(1, at=xat, labels=xlab)

#Add slash to designate axis break
axis.break(1, nmds.xy[3,1]+0.6, brw=0.05, style="slash")
````

````
#EXAMPLE: plot with smaller inset plot in upper right
#Basic plot
plot(nmds, type="n")
points(nmds, pch=20, cex=2.5, display="sites", col=var$col)

#Define smaller area for inset plot
par(fig=c(3/6,2/3,1/6,1/3), new=TRUE)

#Basic inset plot
#EXAMPLE: only use samples 4 through  27
plot(nmds.xy[4:27,1], nmds.xy[4:27,2], pch=20, cex=2.5, col=var$col[4:27])
```

```{r Assess differences in dispersion in nMDS plots: PERMDISP}
#Load package
library(vegan)

#Calculate Bray-Curtis dissimilarity
dist = vegdist(OTU, method="bray")

#Calculate dispersions of age groups
disp = betadisper(dist, var$AgeGroup)

#Test for differences between dispersions of groups (PERMDISP)
permutest(disp, pairwise=TRUE, permutations=10000)
```

```{r Model change in log abundances and log variances}
#Load packages
library(vegan)
library(lme4)
library(car)

#Define data frames and variables. num=number of animals in the comparison
OTU = OTU
var = var
time1 = "1yr"
time2 = "2yr"
num=15

#Make data frame for only samples from time 1. Add a number smaller than the min non-zero abundance
(which is 1)
t1 = OTU[Met$AgeGroup == time1, ]
t1[t1 == 0] = .1

#Repeat for time 2.
t2 = OTU[Met$AgeGroup == time2, ]
t2[t2 == 0] = .1

#Remove any samples that do not have both time 1 and 2 samples, if necessary
````

~~~
t1 = t1[!rownames(t1) %in% c(), ]
t2 = t2[!rownames(t2) %in% c(), ]
Met=Met[!rownames(Met) %in% c(), ]

#Make a matrix of log ratios between time 1 and 2
lr1.2 = as.matrix(log(t2/t1))
hist(lr1.2[, 1:100])

#Make a matrix that corrects the change in abundance by the number of days elapsed
days.elapsed1.2 = Met[Met$AgeGroup == time2, ]$AgeExact - Met[Met$AgeGroup == time1,
]$AgeExact
lr1.2c = sweep(lr1.2, 1, days.elapsed1.2, "/")
hist(lr1.2c[, 1:100])

#create data frame for samples from time 1 and time 2
t1.2 = rbind(t1, t2)
plot(log(apply(t1.2, 2, mean)), log(apply(t1.2, 2, var)))

#Find which OTUs in the merged data frame are above the cutoff equal of presence in at least 2 samples
t1.2.cutoff = (0.1*(num-2)+2)/num
which.t1.2.cutoff = which(apply(t1.2, 2, mean) > t1.2.cutoff)
t1.2.cut = t1.2[, which.t1.2.cutoff]
t1.cut = t1[, which.t1.2.cutoff]
t2.cut = t2[, which.t1.2.cutoff]
lr.t1.2c.cut = lr1.2c[, which.t1.2.cutoff]

#Use the general rule that the log variance scales with log mean abundance to get a measurement of
variability of OTUs
plot(log(apply(t1.2.cut, 2, mean)), log(apply(t1.2.cut, 2, var)))
tay.lm.t1.2 = summary(lm(log(apply(t1.2.cut, 2, var)) ~ log(apply(t1.2.cut, 2, mean))))
abline(tay.lm.t1.2, lwd = 2)
```

```{r Test for differences in community structure (Bray-Curtis) and composition (Jaccard) between
groups: ANOSIM}
#Load package
library(vegan)

#Calculate Bray-Curtis distance matrix
dist = vegdist(OTU, method="bray")

#Perform ANOSIM for age groups
anosim(dist, var$AgeGroup, permutations=10000)
```

```{r Identify OTUs important in significant ANOSIMs: SIMPER}
#Load package
library(vegan)

#Run SIMPER
simper(OTU, var$AgeGroup, permutations = 10000)
~~~

````
```

```{r Determine co-occurrences of OTUs of different amplicons: Mantel}
#Load package
library(vegan)

#Calculate distance matrices for OTU tables. For example, 1 = archaea, 2 = bacteria, 3 = fungi
dist1 = vegdist(OTU)
dist2 = vegdist(OTU2)
dist3 = vegdist(OTU3)

#Run co-variance (Mantel)
#For 2 tables
mantel(dist1, dist2, permutations=10000)
#For 3 tables
mantel.partial(dist1, dist2, dist3, permutations=10000)
```

```{r Assess differences in community structure (Bray-Curtis) and composition (Jaccard) across a continuous variable: PERMANOVA}
#All such tests occurred within age groups
#Subset the OTU table to only 2 week samples
OTU2w = OTU[var$AgeGroup == "2w", ]

#Run PERMANOVA between 2 week OTU table and ADG
adonis(OTU2w~ADG, var[var$AgeGroup=="2w",], permutations = 10000, method="bray")
```

```{r Calculate correlations between OTUs or between OTUs and variables: Kendall}
#Run Kendall correlation between two sets of OTUs
cor.OTU1.OTU2 = cor(OTU1, OTU2, method="kendall")

#Run Kendall correlations between OTUs and a continuous variable
cor.OTU.ADG = cor(OTUs, var$ADG, method="kendall")

#Print results to a text file
write.table(cor.OTU.ADG, file="cor.OTU.ADG.txt", sep="\t")
```
````
````
